# Two Components of the RNA-Directed DNA Methylation Pathway Associate with MORC6 and Silence Loci Targeted by MORC6 in *Arabidopsis*

**DOI:** 10.1371/journal.pgen.1006026

**Published:** 2016-05-12

**Authors:** Zhang-Wei Liu, Jin-Xing Zhou, Huan-Wei Huang, Yong-Qiang Li, Chang-Rong Shao, Lin Li, Tao Cai, She Chen, Xin-Jian He

**Affiliations:** 1 College of Biological Sciences, China Agricultural University, Beijing, China; 2 National Institute of Biological Sciences, Beijing, China; Gregor Mendel Institute of Molecular Plant Biology, AUSTRIA

## Abstract

The SU(VAR)3-9 homolog SUVH9 and the double-stranded RNA-binding protein IDN2 were thought to be components of an RNA-directed DNA methylation (RdDM) pathway in *Arabidopsis*. We previously found that SUVH9 interacts with MORC6 but how the interaction contributes to transcriptional silencing remains elusive. Here, our genetic analysis indicates that SUVH2 and SUVH9 can either act in the same pathway as MORC6 or act synergistically with MORC6 to mediate transcriptional silencing. Moreover, we demonstrate that IDN2 interacts with MORC6 and mediates the silencing of a subset of MORC6 target loci. Like SUVH2, SUVH9, and IDN2, other RdDM components including Pol IV, Pol V, RDR2, and DRM2 are also required for transcriptional silencing at a subset of MORC6 target loci. MORC6 was previously shown to mediate transcriptional silencing through heterochromatin condensation. We demonstrate that the SWI/SNF chromatin-remodeling complex components SWI3B, SWI3C, and SWI3D interact with MORC6 as well as with SUVH9 and then mediate transcriptional silencing. These results suggest that the RdDM components are involved not only in DNA methylation but also in MORC6-mediated heterochromatin condensation. This study illustrates how DNA methylation is linked to heterochromatin condensation and thereby enhances transcriptional silencing at methylated genomic regions.

## Author Summary

DNA methylation is a conserved epigenetic mark that is required for the silencing of transposons and introduced transgenes in eukaryotes. An RNA-directed DNA methylation pathway mediates *de novo* DNA methylation and thereby leads to transcriptional silencing in *Arabidopsis*. In this study, we find that two RNA-directed DNA methylation components interact with the microrchidia (MORC) protein MORC6 and lead to transcriptional silencing through a mechanism that is distinct from the RNA-directed DNA methylation pathway. MORC6 was previously thought to mediate transcriptional silencing through heterochromatin condensation. Our study suggests that the interaction of the RNA-directed DNA methylation components with MORC6 may mediate a link between DNA methylation and heterochromatin condensation.

## Introduction

DNA methylation is an important chromatin mark related to transposon silencing, gene imprinting, genome stability, and gene expression regulation [1–3]. In *Arabidopsis*, the DNMT1-like DNA methyltransferase MET1 is the main enzyme responsible for maintaining DNA methylation at symmetric CG sites [4,5]. The SNF2-type chromatin-remodeling protein DDM1 facilitates the maintenance of DNA methylation [6,7]. CMT3is a plant-specific DNA methyltransferase that is mainly responsible for maintenance of DNA methylation at CHG sites (H is A, T, or C) [5,8]. Unlike in animals, DNA methylation at asymmetric CHH sites is widespread in plants [1,9]. In *Arabidopsis*, CMT2, another plant-specific DNA methyltransferase, is required for the maintenance of CHH methylation at heterochromatin regions [7,10]. DRM2 is a DNMT3-like *de novo* DNA methyltransferase that is responsible for establishing DNA methylation at CHH sites and to a lesser extent at CG and CHG sites [5,11].

DNA methylation mediated by DRM2 requires an RNA-directed DNA methylation (RdDM) pathway, which has been well studied in the past decade [1,12,13]. Two atypical multi-subunit DNA-dependent RNA polymerases, Pol IV and Pol V, produce noncoding RNAs at RdDM target loci [14–18]. The RNA-dependent RNA polymerase RDR2 associates with Pol IV and is required for the conversion of single-stranded RNAs produced by Pol IV into double-stranded RNAs [14]. The Dicer-like protein DCL3 acts as an RNase to cleave the double-stranded RNAs into 24-nucleotide (nt) small interfering RNAs (siRNAs), which are loaded onto AGO4 and form an AGO4-siRNA complex required for DNA methylation [19–21]. Noncoding RNAs produced by Pol V are thought to function as scaffold RNAs that recruit AGO4-siRNA to chromatin [22]. IDN2, a double-stranded RNA-binding protein, interacts with its paralogs IDP1 and IDP2, forming a heteromer required for RdDM [23–26]. Pol V-produced scaffold RNAs are bound not only by AGO4 but also by IDN2 and DRM2. AGO4 and IDN2 are required for the binding of DRM2 to Pol V-produced scaffold RNAs [27]. KTF1/SPT5L functions together with AGO4 and acts as an effector in the RdDM pathway [28,29]. SHH1/DTF1 binds to histone H3K9 methylation by its SAWADEE domain and mediates the recruitment of Pol IV to chromatin at a subset of RdDM target loci [30,31]. DMS3, DRD1, and RDM1 are three important components in the RdDM pathway [32–34] and form a DDR complex required for Pol V occupancy on chromatin [17,35,36]. SUVH2 and SUVH9, which belong to the SRA domain-containing SU(VAR)3-9 protein family, bind to methylated DNA by their SRA domains and associate with the DDR complex to mediate the recruitment of Pol V to RdDM target loci [37–39].

The *Arabidopsis* microrchidia (MORC) ATPase family proteins are conserved among plants and animals and are involved in transcriptional silencing [40–42]. In *Arabidopsis*, MORC6/DMS11 was identified by different forward genetic screens as a regulator of transcriptional silencing [40,41,43]. The silencing of some well-known RdDM target loci is affected in the *morc6*/*dms11* mutants [41,43], suggesting a functional connection between MORC6 and RdDM in transcriptional silencing. In the *morc6*/*dms11* mutants, however, DNA methylation is either weakly reduced or not affected at RdDM target loci [40,41,43], indicating that MORC6 is unlikely to be a canonical RdDM component. MORC6 was reported to function in the condensation of pericentromeric heterochromatin, thereby facilitating transcriptional silencing [40]. Recent studies demonstrated that MORC6 and its homologs MORC1 and MORC2form a complex [39,44]. The MORC family proteins (MORCs) physically associate with the recently characterized RdDM component SUVH9 [39], indicating that the involvement of MORCs in transcriptional silencing is likely related to the RdDM pathway. However, the molecular mechanism underlying the function of MORCs in transcriptional silencing is elusive.

In addition to DNA methylation, chromatin structure affects transcription throughout the genome [45]. An octamer of histone proteins is wrapped by 147-bp DNA and forms a nucleosome core particle on chromatin [46]. ATP-dependent chromatin-remodeling proteins are required to change chromatin structure by altering the positions of nucleosomes, thus regulating the transcription status of the corresponding chromatin loci [47–49]. The *Arabidopsis* genome encodes41 SNF2-related chromatin-remodeling proteins, among which BRM and SYD act as ATP-driven motor subunits of the SWI/SNF-type chromatin-remodeling complexes [50]. SWI3 subunits are essential subunits of the SWI/SNF chromatin-remodeling complexes and play important roles during plant development [51]. SWI3B, a subunit of the SWI/SNF complex, physically interacts with IDN2, an RdDM component that binds to Pol V-produced noncoding RNAs, and mediates transcriptional silencing by nucleosome positioning [52]. The SNF2-related chromatin-remodeling proteins CHR19, CHR27/FRG1, and CHR28/FRG2 contribute to nucleosome positioning and transcriptional silencing at RdDM target loci by associating with the SU(VAR)3-9 family protein SUVR2 [53,54]. These results suggest that proper nucleosome positioning mediated by these chromatin-remodeling proteins is responsible for maintaining chromatin in a transcriptionally repressive status. It remains unclear, however, how the chromatin-remodeling proteins are targeted to repressive chromatin regions and contribute to transcriptional silencing.

In the RdDM pathway, SUVH2/9 were thought to bind methylated DNA and then recruit Pol V to chromatin [38,39], whereas IDN2 binds to Pol V-produced noncoding RNA and thereby facilitate DNA methylation and transcriptional silencing [27,52]. In this study, we demonstrate that the RdDM component IDN2 and the SWI/SNF chromatin-remodeling complex components interact with MORC6 and are involved in transcriptional silencing at a subset of MORC6 target loci. Moreover, we demonstrate that the RdDM components including SUVH2/9, Pol IV, Pol V, RDR2, and DRM2 are required for transcriptional silencing at some MORC6 target loci. The interaction of SUVH2/9 with MORC6 was already reported in our previous study [39]. We propose that the RdDM components SUVH2/9 and IDN2 cooperate with MORC6 and play critical roles in transcriptional silencing that are different from their roles in the RdDM pathway.

## Results

### MORC6 contributes to DNA methylation at a small subset of RNA-directed DNA methylation target loci

Although previous studies have demonstrated that the *Arabidopsis* MORC family protein MORC6 mediates transcriptional silencing [40,41,43], how MORC6 mediates transcriptional silencing remains to be determined. Previous studies indicated that MORC6 physically interacts with SUVH9, which functions redundantly with its homolog SUVH2 in recruiting Pol V to methylated chromatin regions and thereby mediates DNA methylation in the RdDM pathway [38,39]. The interaction of MORC6 with SUVH9 suggests that MORC6 may be functionally related to the RdDM pathway.

**Fig 1:**
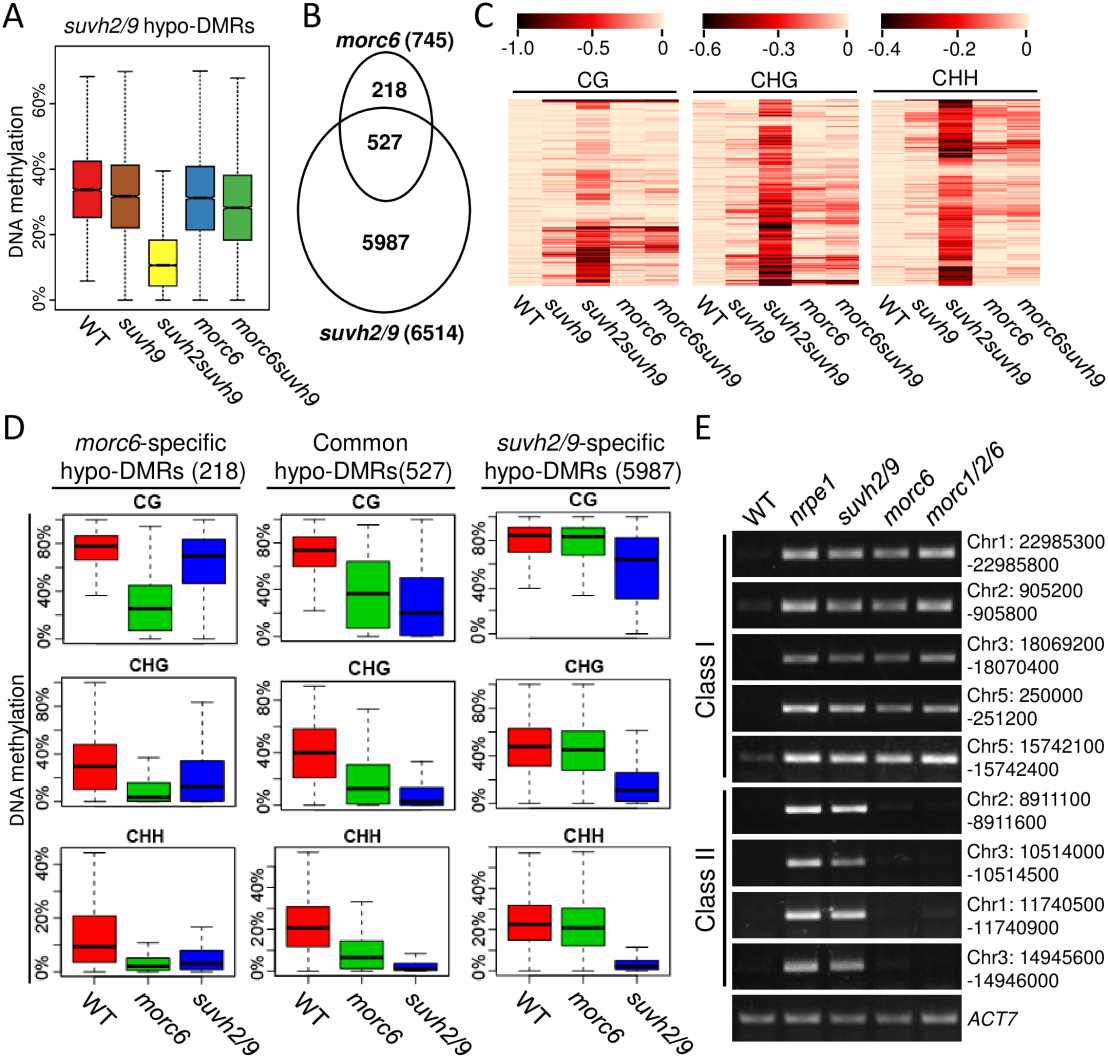
The involvement of MORC6 in DNA methylation at a small subset of RdDM target loci. (A) Box plots showing DNA methylation levels of *suvh2/9* hypo-DMRs in the indicated mutants. (B) Venn diagram of overlap between hypo-DMRs in *morc6* and *suvh2/9*. (C) Heat maps showing DNA methylation changes within *suvh2/9* hypo-DMRs in the wild type and the mutants. Different scales were used for CG, CHG, and CHH methylation. In the scales, the DNA methylation levels of the wild type were set as 0 (light yellow lines) and absolute DNA methylation changes were represented by lines whose colors are ranged from light yellow to black. (D) DNA methylation levels of *morc6*-specific hypo-DMRs, *suvh2/9*-specific hypo-DMRs, and hypo-DMRs shared by *morc6* and *suvh2/9* are indicated at three cytosine contexts in the wild type, *morc6*, and *suvh2/9*. (E) Validation of bisulfite sequencing results by PCR-based DNA methylation analysis. Genomic DNA was cleaved by McrBC, a restriction enzyme that specifically recognizes methylated DNA. Class I represents hypo-DMRs shared by *morc6* and *suvh2/9*; Class II represents *suvh2/9*-specific hypo-DMRs. The RdDM pathway contributes to CHH methylation and to a lesser extent to CG and CHG methylation [5], but it is unknown how MORC6 contributes to DNA methylation at the three cytosine contexts. Our analysis indicated that *morc6* affects DNA methylation at all the three cytosine contexts (CG, CHG, and CHH) in a small subset of the 6514 *suvh2/9* hypo-DMRs (Fig 1C). In the 527 hypo-DMRs shared in *morc6* and *suvh2/9*, DNA methylation is reduced at all three cytosine contexts in both *morc6* and *suvh2/9* (Fig 1D). In the 218 *morc6* specific hypo-DMRs, DNA methylation is reduced at all three cytosine contexts in the *morc6* mutant (Fig 1D). Although the 218 *morc6* specific hypo-DMRs do not overlap with the *suvh2/9* hypo-DMRs, the DNA methylation level of the 218 hypo-DMRs is reduced at CHG and CHH sites in *suvh2/9* (Fig 1D). However, CG methylation of the 218 hypo-DMRs is markedly reduced in *morc6* but not in *suvh2/9* (Fig 1D). Thus, these 218 hypo-DMRs could be either RdDM-independent loci or RdDM-dependent loci that are not dependent on SUVH2/9.

To understand how MORC6 functions in transcriptional silencing, we assessed whole-genome DNA methylation of the wild type, *suvh9*, *suvh2/9*, *morc6*, and *morc6suvh9* by bisulfite sequencing analysis. The analysis identified 6514 hypo-differentially methylated regions (hypo-DMRs) in *suvh2/9* (S1 and S2 Tables). Our small RNA deep sequencing data indicated that Pol IV-dependent 24-nt siRNAs are enriched in most hypo-DMRs identified in *suvh2/9* (S2 Table). The results are consistent with the involvement of SUVH2/9 in the RdDM pathway [38,39]. We thereafter took the hypo-DMRs identified in *suvh2/9* as RdDM target loci in this study. Box plots were used to indicate the DNA methylation levels of the 6514 SUVH2/9 target loci in different genotypes. The result indicated that the overall DNA methylation levels of these loci are not significantly affected in *morc6* (Fig 1A), which is in agreement with a previous report [40]. Moreover, we found that the overall DNA methylation levels of the SUVH2/9 target loci are not significantly reduced in the *suvh9* single mutant (Fig 1A), which supports the notion that SUVH9 is functionally redundant with its homolog SUVH2 in DNA methylation [37,55]. DNA methylation is slightly reduced in the *morc6suvh9* double mutant compared with the *suvh9* single mutant and the wild type (Fig 1A), suggesting that MORC6 may weakly contribute to DNA methylation at RdDM target loci. Our analysis identified 745 hypo-DMRs and 302 hyper-DMRs in *morc6* relative to the wild type (S1 and S3 Tables). In *morc6*, the number of hypo-DMRs is clearly higher than that of hyper-DMRs, even though the number of hypo-DMRs is much lower in *morc6* than in *suvh2/9*. Our small RNA deep sequencing data suggest that Pol IV-dependent 24-nt siRNAs are also enriched in most hypo-DMRs identified in *morc6* (S3 Table), suggesting a possible role of MORC6 in the RdDM pathway. Most of the 745 hypo-DMRs (527/745) in *morc6* overlap with the hypo-DMRs in *suvh2/9* (Fig 1B), a degree of overlap that is markedly higher than expected by chance (p<0.01, hypergeometric test). The hypo-DMRs identified in both *suvh2/9* and *morc6* are enriched in annotated TEs and intergenic regions rather than in genes (S1 Fig), which is consistent with the function of SUVH2/9 and MORC6 in silencing of TEs and intergenic noncoding regions. These analyses suggest that MORC6 contributes to DNA methylation at a small subset (Class I: 527 loci) of SUVH2/9 target loci, whereas MORC6 is dispensable for DNA methylation at most (Class II: 5987 loci) of SUVH2/9 target loci (Fig 1B).

To confirm the effect of *morc6* on DNA methylation as determined by the whole-genome DNA methylation analysis, we randomly selected five loci from the 527 overlapping hypo-DMRs (Class I) in *morc6* and *suvh2/9* for validation (Figs 1E and S2A; S2 and S3 Tables). McrBC is a restriction enzyme that specifically cleaves methylated DNA. We used McrBC to cleave genomic DNA and then performed a PCR-based DNA methylation assay. The result indicated that the DNA methylation levels of the five class I loci are reduced in *morc6* as well as in the RdDM mutants *nrpe1* and *suvh2/9* (Fig 1E), confirming that MORC6 functions in DNA methylation at a small subset of RdDM target loci. Furthermore, we randomly selected four loci from the 5987 *suvh2/9*-specific hypo-DMRs (Class II) for validation (Figs 1E and S2B; S2 Table). The result showed that the DNA methylation levels of the four class II loci are reduced in *nrpe1* and *suvh2/9* but not in *morc6* (Fig 1E), demonstrating that MORC6 is dispensable for DNA methylation at majority of RdDM target loci. Because MORC6 forms a complex with its two close homologs, MORC1 and MORC2 [39,44], MORC6 and its homologs may function redundantly in DNA methylation at these class II hypo-DMRs. We generated a *morc1* mutation by the CRISPR/CAS9 system (S3 Fig), and then introduced the mutation into a *morc2/6* double mutant by crossing to get a *morc1/2/6* triple mutant. We determined whether DNA methylation of the four class II loci are affected in the triple mutant. Our result indicated, however, that DNA methylation is not reduced at the four loci even in the *morc1/2/6* triple mutant (Fig 1E). We further determined the effect of *morc6* and *morc1/2/6* on DNA methylation at the known RdDM target loci *solo LTR*, *AtSN1*, an *ETR7*. The DNA methylation levels of the loci are reduced in *nrpe1* and *suvh2/9* but not in *morc6* and *morc1/2/6* (S4 Fig). We used *TSI*, a highly methylated locus, as a control, and demonstrated that McrBC works equivalently in the wild type, *nrpe1*, *suvh2/9*, *morc6*, and *morc1/2/6* (S5 Fig). These results suggest that the MORC family proteins contribute to DNA methylation only at a small subset of RdDM target loci.

### SUVH2/9 and MORC6 contribute to transcriptional silencing through the same genetic pathway at a subset of their common target TEs

**Fig 2:**
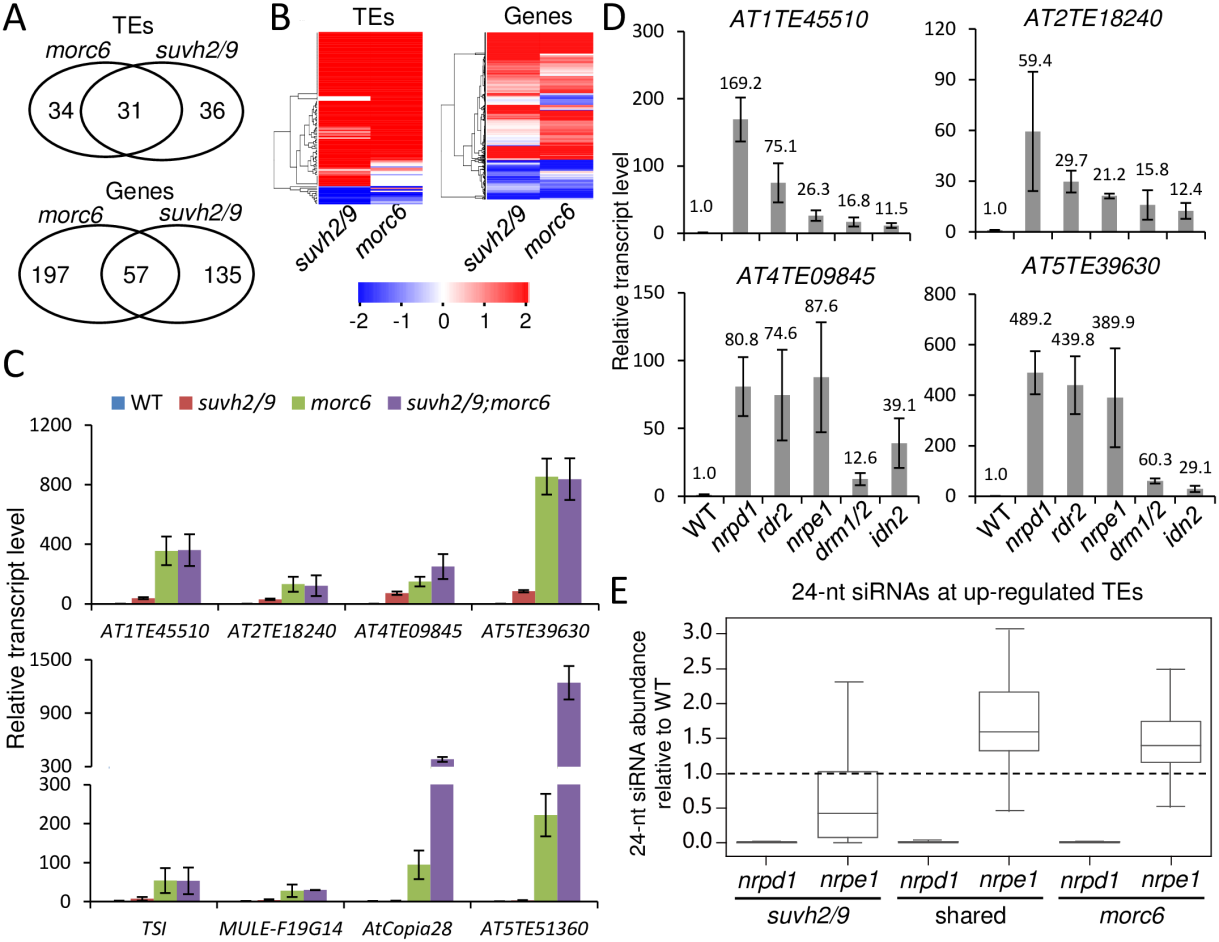
Identification and characterization of target loci shared by SUVH2/9 and MORC6. (A) Venn diagrams of overlap between up-regulated (2-fold increase; p<0.01 by Cufflinks) TEs and genes in *morc6* and *suvh2/9*. (B) Heat maps of differentially expressed TEs and genes in *suvh2/9* and *morc6* relative to the wild type. Up- and down-regulated loci are shown by red and blue lines, respectively. Color scales represent log_2_ (mutant/WT). (C) The RNA transcript levels of MORC6 target loci were determined by quantitative RT-PCR in the wild type, *suvh2/9*, *morc6*, and *suvh2/9;morc6*. *ACT2* was used as an internal control. Error bars are standard deviation of three biological replicates. (D) Effect of the RdDM mutations *nrpd1*, *rdr2*, *nrpe1*, *drm1/2*, and *idn2* on the transcript levels of SUVH2/9 and MORC6 common target TEs as determined by quantitative RT-PCR analysis. Error bars are standard deviation of three biological replicates. (E) The effect of *nrpd1* and *nrpe1* on the abundance of 24-nt siRNAs at SUVH2/9 and MORC6 target TEs. Based on small RNA deep sequencing data, the 24-nt siRNA abundance in *nrpd1* and *nrpe1* is compared with that in the wild type and fold changes are indicated by y-axis.

We carried out RNA-sequencing (RNA-seq) analysis to compare the effect of *suvh2/9* and *morc6* on transcriptional silencing. The RNA-seq data showed that 67 and 65 transposable elements (TEs) are significantly (fold-change>2; p<0.01 by Cufflinks) up-regulated in *suvh2/9* and *morc6*, respectively, whereas only 10 and 4 TEs are significantly down-regulated in *suvh2/9* and *morc6*, respectively (S4 Table). The result is consistent with the function of SUVH2/9 and MORC6 in TE silencing as previously reported [37,40,41]. We found that 31 up-regulated TEs are common to *suvh2/9* and *morc6* (Fig 2A), which are thought to be common target TEs of SUVH2/9 and MORC6. The numbers of overlapping up-regulated TEs are significantly higher than expected by chance (p<0.01, hypergeometric test). The effect of *suvh2/9* and *morc6* on the transcript levels of genes was also determined by the RNA-seq analysis. The result showed that 192 and 254 genes are significantly (fold-change>2; p<0.01 by Cufflinks) up-regulated in *suvh2/9* and *morc6*, respectively (Fig 2A; S5 Table). Fifty-seven genes are co-up-regulated in *suvh2/9* and *morc6* (Fig 2A), which are thought to be common target genes of SUVH2/9 and MORC6. The numbers of the co-up-regulated genes are also significantly higher than expected by chance (p<0.01, hypergeometric test). Heat maps indicated that the transcript levels of TEs and genes tend to be co-regulated in *suvh2/9* and *morc6* (Fig 2B). These results suggest that SUVH2/9 and MORC6 are functionally connected in transcriptional silencing.

To confirm the RNA-seq data, we randomly selected four co-up-regulated TEs including *AT1TE45510*, *AT2TE18240*, *AT4TE09845*, and *AT5TE39630* for validation by quantitative RT-PCR analysis. The result confirmed that the transcriptional levels of all the four TEs are significantly up-regulated in *suvh2/9* and *morc6* (Fig 2C). To determine the genetic relationship between *suvh2/9* and *morc6*, we generated a *suvh2/9;morc6* triple mutant by crossing and examined how the transcript levels of the SUVH2/9 and MORC6 common target TEs are affected in the *suvh2/9;morc6* triple mutant. By quantitative RT-PCR analysis, we found that the transcript levels are not significantly enhanced for *AT1TE45510*, *At2TE18240*, *AT4TE09845*, and *AT5TE39630* in *suvh2/9;morc6* relative to either *suvh2/9* or *morc6* (Fig 2C). The results suggest that SUVH2/9 and MORC6 function in the same genetic pathway at these target loci. To comprehensively understand the relationship between MORC6 and SUVH2/9, we also analyzed the transcript levels of loci that are specifically targeted by MORC6 (Fig 2C). The silencing of the MORC6 specific target loci including *TSI*, *MULE-F19G14*, *AtCopia28*, *AT5TE51360* is markedly released in *morc6* and is either not released or slightly released in *suvh2/9* (Fig 2C). We found that the silencing of *AtCopia28* and *AT5TE51360* is markedly enhanced in *suvh2/9;morc6* relative to *morc6* (Fig 2C), suggesting that SUVH2/9 act synergistically with MORC6 in transcriptional silencing at these loci even though disruption of SUVH2/9 in the wild-type background only slightly affect the silencing of these loci. These results suggest that SUVH2/9 act in the same pathway as MORC6 in a subset of MORC6 target loci and act synergistically with MORC6 in some other MORC6 target loci.

### Core RNA-directed DNA methylation components are involved in the silencing of SUVH2/9 and MORC6 common target loci

SUVH2/9 bind to methylated DNA and thereby mediate the recruitment of Pol V to chromatin in the RdDM pathway [38,39]. Thus, we expect that core components of the RdDM pathway may be involved in the silencing of SUVH2/9 and MORC6 target loci. By quantitative RT-PCR analysis, we found that the transcript levels of the SUVH2/9 and MORC6 common target loci *AT1TE45510*, *AT2TE18240*, *AT4TE09845*, and *AT5TE39630* are markedly increased in *nrpd1*, *rdr2*, *nrpe1*, *drm1/2*, and *idn2* relative to the wild type (Fig 2D), suggesting that the RdDM components NRPD1, RDR2, NRPE1, DRM1/2, and IDN2 are required for the silencing of the SUVH2/9 and MORC6 target loci.

NRPD1 and RDR2 are responsible for producing precursors of Pol IV-dependent 24-nt siRNAs [14,18]. Pol IV-dependent 24-nt siRNAs are highly enriched in TEs and other DNA repeats throughout the genome [56,57]. Thus, it is possible that Pol IV-dependent 24-nt siRNAs are probably associated with the silencing of SUVH2/9 and MORC6 common target loci. We used our small RNA deep sequencing data to analyze the accumulation of 24-nt siRNAs and found that Pol IV-dependent 24-nt siRNAs are enriched in most of SUVH2/9 and MORC6 common target loci (Fig 2E; S6 and S7 Tables), suggesting that Pol IV-dependent 24-nt siRNAs associate with in the silencing of SUVH2/9 and MORC6 target loci.

Pol V is indirectly involved in 24-nt siRNA accumulation at a subset of Pol IV-dependent 24-nt siRNA loci [56,58]. Pol IV- and Pol V-dependent siRNAs are predominantly present at dispersed TEs and DNA repeats across the chromosome arms, whereas Pol IV-dependent and Pol V-independent siRNAs tend to appear at highly duplicated TEs and other DNA repeats in pericentromeric heterochromatin regions [56,58]. Our analysis indicated that the Pol V largest subunit NRPE1 is required for the accumulation of Pol IV-dependent siRNAs at SUVH2/9-specific target TEs but not at SUVH2/9 and MORC6 common target TEs or MORC6-specific target TEs (Fig 2E). The enrichment of Pol IV-dependent and Pol V-independent siRNAs on SUVH2/9 and MORC6 common target TEs is consistent with the previous report that MORC6 mainly functions in pericentromeric heterochromatin regions [40].

### MORC6 and SUVH2/9 contribute to transcriptional silencing without alteration in DNA methylation

**Fig 3:**
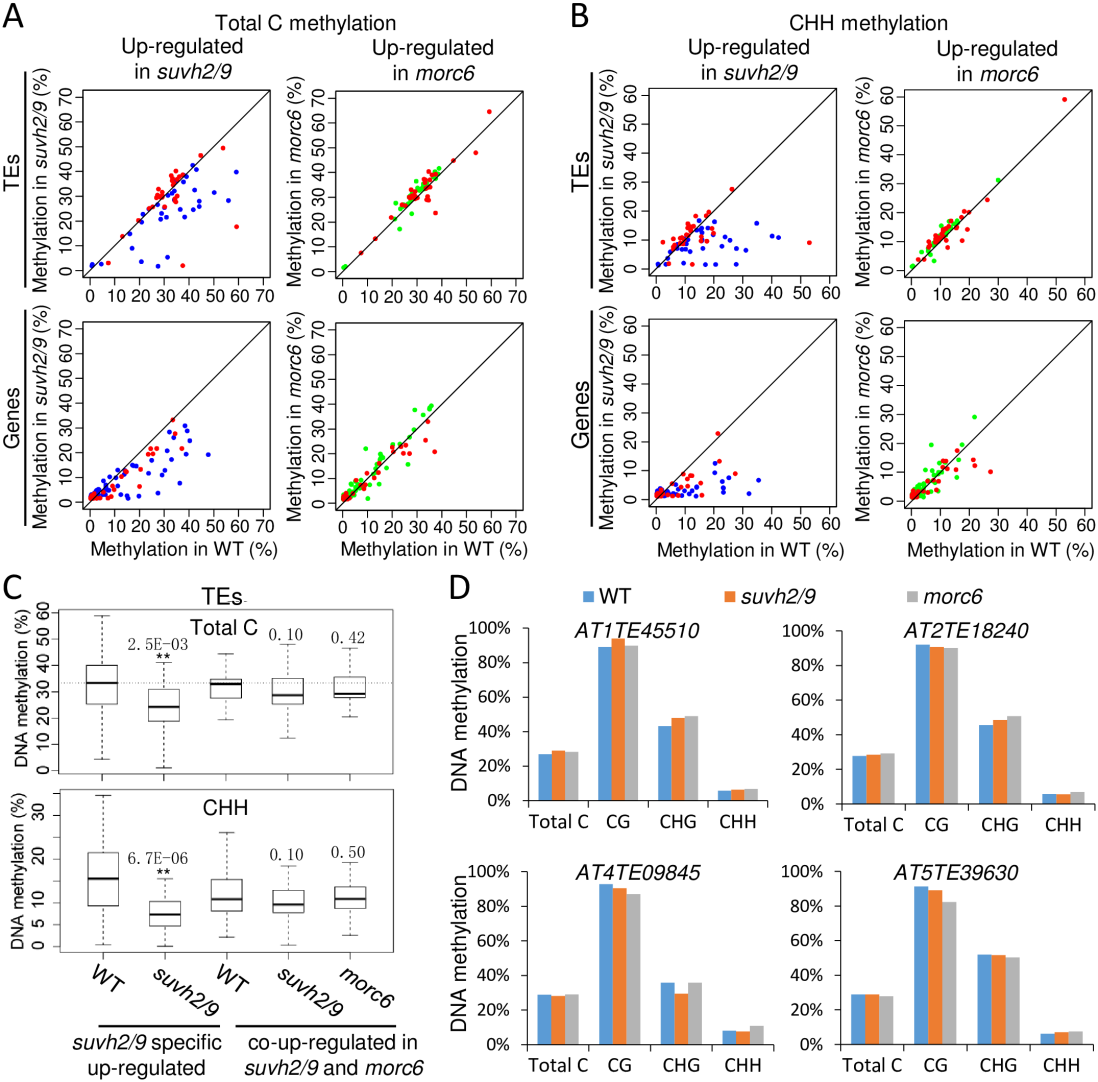
Determination of effect of *suvh2/9* and *morc6* on DNA methylation of SUVH2/9 and MORC6 target loci. (A, B) Scatter plots showing DNA methylation of transcriptionally up-regulated TEs and genes in *suvh2/9* and *morc6*. Red dots represent TEs and genes that are co-up-regulated in *suvh2/9* and *morc6*. Blue dots represent TEs and genes that are up-regulated in *suvh2/9* but not in *morc6*. Green dots represent TEs and genes that are up-regulated in *morc6* but not in *suvh2/9*. (C) Box plots showing DNA methylation of transcriptionally up-regulated TEs in *morc6* and *suvh2/9* at total cytosine sites (top panel) and CHH sites (bottom panel). DNA methylation of *suvh2/9*-specific up-regulated TEs is indicated in the wild type and *suvh2/9*, and DNA methylation of co-up-regulated TEs in *suvh2/9* and *morc6* is indicated in the wild type, *suvh2/9*, and *morc6*. Asterisks indicate statistical significance (* p<0.05, ** p<0.01; t-test). p values are shown on top of bars. (D) Effect of *suvh2/9* and *morc6* on DNA methylation of *AT1TE45510*, *AT2TE18240*, *AT4TE09845*, and *AT5TE39630* at total cytosine, CG, CHG, and CHH sites. The DNA methylation data were generated from the whole-genome bisulfite sequencing analysis in the wild type, *suvh2/9*, and *morc6*.

To understand how SUVH2/9 and MORC6 contribute to transcriptional silencing, we determined whether DNA methylation is affected by *suvh2/9* and *morc6* at SUVH2/9 and MORC6 target loci. Analysis of the whole-genome bisulfite sequencing data indicated that the overall DNA methylation level of SUVH2/9 target TEs is lower in *suvh2/9* than in the wild type (S6 Fig; S6 Table). In a subset of SUVH2/9 target TEs, DNA methylation is reduced by *suvh2/9* (Figs 3A–3C and S7A–S7D; S6 Table), supporting a role of SUVH2/9 in the RdDM pathway. In the other subset of SUVH2/9 target TEs, however, DNA methylation is not significantly reduced by *suvh2/9* in all the three cytosine contexts CG, CHG, and CHH (Figs 3A–3C and S7A–S7D; S6 Table), suggesting that SUVH2/9 mediate the silencing of these TEs by a mechanism that is different from the RdDM pathway. In *morc6*, DNA methylation is not significantly reduced at MORC6 target TEs in all the three cytosine contexts (Figs 3A–3C and S7A–S7D; S6 Table), demonstrating that the involvement of MORC6 in transcriptional silencing is independent of alteration in DNA methylation.

Our RNA-seq analysis identified 31 TEs that are co-up-regulated in *suvh2/9* and *morc6* (Fig 2A). As determined by our whole-genome DNA methylation analysis, the DNA methylation levels of most of the co-up-regulated TEs are not significantly reduced in *suvh2/9* as well as in *morc6* (Figs 3A–3C and S7A–S7D; S6 Table). We performed quantitative RT-PCR analysis to determine the transcript levels of four randomly selected co-up-regulated TEs (*AT1TE45510*, *AT2TE18240*, *AT4TE09845*, and *AT5TE39630*). The result confirmed that the transcript levels of all the four TEs are co-up-regulated in *suvh2/9* and *morc6* (Fig 2C). The whole-genome DNA methylation data indicated that the DNA methylation levels of the four co-up-regulated TEs are not reduced in *suvh2/9* and *morc6* (Fig 3D). We randomly selected three co-up-regulated TEs to determine their DNA methylation levels by locus-specific bisulfite sequencing analysis. The result confirmed that the DNA methylation levels of the three TEs are not reduced in *suvh2/9* and *morc6* (S8 Fig). These results suggest that DNA methylation change is not required for the function of SUVH2/9 in transcriptional silencing at SUVH2/9 and MORC6 common target TEs.

When DNA methylation is present in the promoter regions of protein-coding genes, it usually causes transcriptional silencing of these genes. Therefore, we determined whether the effect of *suvh2/9* and *morc6* on the up-regulation of genes is correlated with the reduction of DNA methylation in the promoter regions. Our whole-genome bisulfite sequencing data suggested that among the 192 up-regulated genes in *suvh2/9*, 53 are significantly methylated (>5%) at the promoter regions in the wild type (S7 Table). In most of the 53 genes, the promoter DNA methylation is lower in *suvh2/9* than in the wild type especially at CHG and CHH sites (Figs 3A, 3B, S6, S7A and S7C; S7 Table), which is consistent with the function of SUVH2/9 in RdDM. Among the 254 up-regulated genes in *morc6*, 75 are significantly methylated (>5%) at the promoter regions in the wild type (S7 Table). The promoter methylation levels for most of the up-regulated methylated genes in *morc6*, however, are similar to that in the wild type at all the three cytosine contexts (Figs 3A, 3B, S6, S7A and S7C; S7 Table), indicating that MORC6 is dispensable for DNA methylation even though it is necessary for transcriptional silencing. Although the promoter DNA methylation levels are reduced in *suvh2/9* for most of SUVH2/9 target genes, the reduction of DNA methylation in *suvh2/9* is much weaker for SUVH2/9 and MORC6 common target genes than for SUVH2/9-specific target genes (Figs 3A, 3B, S6, S7A and S7C; S7 Table). These results suggest that SUVH2/9 may cooperate with MORC6 to mediate the silencing of SUVH2/9 and MORC6 common target loci through a mechanism that is different from the RdDM pathway.

### The role of the core RdDM components in DNA methylation of MORC6 target loci

**Fig 4:**
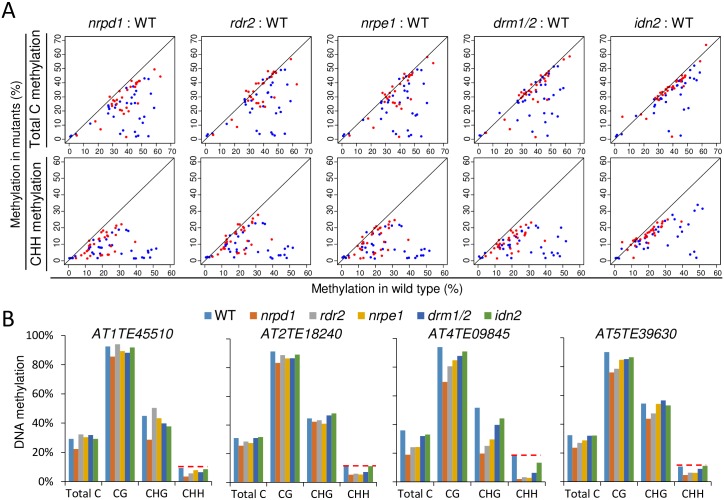
Determination of effect of RdDM mutations on the transcript and DNA methylation levels of SUVH2/9 and MORC6 target TEs. (A) Scatter plots showing DNA methylation of SUVH2/9 and MORC6 target TEs in *nrpd1*, *rdr2*, *nrpe1*, *drm1/2*, and *idn2* relative to the wild type. Red dots represent target TEs that are transcriptionally co-up-regulated in *suvh2/9* and *morc6*, whereas blue dots represent TEs that are transcriptionally up-regulated in *suvh2/9* but not in *morc6*. DNA methylation levels at total cytosine and CHH sites are shown in top and bottom panels, respectively. (B) The DNA methylation levels of the SUVH2/9 and MORC6 common target TEs in the wild type, *nrpd1*, *rdr2*, *nrpe1*, *drm1/2*, *suvh2/9*, and *morc6*. The DNA methylation levels of the TEs are separately shown in total cytosine, CG, CHG, and CHH sites.

We have demonstrated that the core RdDM components NRPD1, RDR2, NRPE1, DRM1/2, and IDN2 are required for the silencing of some SUVH2/9 and MORC6 common target loci (Fig 2D). To understand how the RdDM components contribute to the silencing of the SUVH2/9 and MORC6 common target loci, we determined whether DNA methylation is affected by the RdDM mutations at these loci. Whole-genome bisulfite sequencing data were previously generated for the RdDM mutants including *nrpd1*, *rdr2*, *nrpe1*, *drm1/2*, and *idn2* [5]. We downloaded the data and analyzed the DNA methylation levels of SUVH2/9 target loci in the RdDM mutants. Our analysis indicated that the DNA methylation levels of total cytosine sites are either not reduced or weakly reduced in SUVH2/9 and MORC6 common target TEs and tend to be reduced to a more extent in SUVH2/9-specific target TEs (Figs 4A, S9, S10A and S10B; S6 Table). To further examine the effect of the RdDM mutations on DNA methylation at SUVH2/9 target TEs, we analyzed the DNA methylation levels of these TEs separately in three cytosine contexts: CG, CHG, and CHH. We found that CHH methylation tends to be reduced by the RdDM mutations in SUVH2/9 target TEs and that the reduction of CHH methylation is weaker in SUVH2/9 and MORC6 common target TEs than in SUVH2/9-specific target TEs (Figs 4A, S9, S10A and S10B; S6 Table). The effect of the RdDM mutations on CG and CHG methylation is weaker than that on CHH methylation (S9, S10A and S10B Figs). These results suggest that the RdDM components contribute to DNA methylation of SUVH2/9 target TEs especially at CHH sites and that the function of the RdDM components in DNA methylation is much weaker in SUVH2/9 and MORC6 common target TEs than in SUVH2/9-specific target TEs. We also determined the effect of the RdDM mutations on DNA methylation at the promoter regions of SUVH2/9 target genes and found that the promoter DNA methylation levels of most SUVH2/9 target genes are markedly reduced in the RdDM mutants (S9, S11A and S11B Figs; S7 Table). Similarly, the effect of the RdDM mutations on DNA methylation is weaker in SUVH2/9 and MORC6 common target genes than in SUVH2/9-specific target genes (S9, S11A and S11B Figs; S7 Table).

Our quantitative RT-PCR analysis demonstrated that the silencing of the SUVH2/9 and MORC6 common target TEs *AT1TE45510*, *AT2TE18240*, *AT4TE09845*, and *AT5TE39630* is released in the RdDM mutants *nrpd1*, *rdr2*, *nrpe1*, *drm1/2*, and *idn2* (Fig 2D). We determined whether the release of the four common target TEs in the RdDM mutants is correlated with changes in DNA methylation. From the whole-genome DNA methylation data, we found that CHH methylation of the four TEs is reduced to various extents in the RdDM mutants *nrpd1*, *rdr2*, *nrpe1*, and *drm1/2*, even though CG and CHG methylation is either not reduced or weakly reduced (Fig 4B). In the *idn2* mutant, CHH methylation is reduced in *AT1TE45510* and *AT4TE09845* but not in *AT2TE18240* and *AT5TE39630* (Fig 4B). These results suggest that the core RdDM components may be involved in the silencing of SUVH2/9 and MORC6 common target loci not only through the RdDM pathway but also through a MORC6-dependent silencing pathway.

We further determine whether the DNA methylation levels of MORC6 specific target loci are affected in the RdDM mutants including *nrpd1*, *nrpe1*, *drm1/2*, and *idn2*. The results indicated that although the silencing of these loci is not significantly released in the RdDM mutants, their DNA methylation is reduced to various extents, suggesting that these loci are subjected to RdDM even though the DNA methylation change is not sufficient for release of silencing (S12, S13 and S14 Figs). In these canonical RdDM mutants, the reduction of DNA methylation in MORC6 specific target loci is comparable to that in SUVH2/9 and MORC6 common target loci and is weaker than that in SUVH2/9 specific target loci (S10, S11and S14 Figs; S6 and S7 Tables). The above quantitative RT-PCR analysis indicated that SUVH2/9 act synergistically with MORC6 in transcriptional silencing at some MORC6 specific target loci (Fig 2C). These results suggest that DNA methylation mediated by the RdDM pathway is involved in enhancing transcriptional silencing at a subset of MORC6 target loci.

### MORC6 interacts with SUVH9 and IDN2

In the RdDM pathway, SUVH2 and SUVH9 function redundantly in the recruitment of Pol V to chromatin, thus contributing to Pol V transcription and DNA methylation [38,39]. The interaction of MORC6 with SUVH2/9 suggests that that MORC6 may have the same function as SUVH2/9 in Pol V transcription and DNA methylation. We performed quantitative RT-PCR to determine whether the MORC6 is involved in Pol V transcription. Consistent with previous reports [17,39,59], we found that the Pol V-produced transcripts *IGN5* and *IGN25* are markedly affected in *nrpe1* and *suvh2/9* (S15 Fig). However, the transcript levels of *IGN5* and *IGN25* are either not reduced or are weakly reduced in the *morc6* single mutant as well as in the *morc1/2/6* triple mutant (S15 Fig). Moreover, we have demonstrated that unlike the canonical RdDM components, MORC6 is involved in transcriptional silencing independently of changes in DNA methylation (Figs 2A–2C, 3A–3D, S6, S7 and S8; S6 and S7 Tables). These results suggest that MORC6 is different from the canonical RdDM components and is involved in transcriptional silencing by a mechanism that is distinct from the RdDM pathway.

**Fig 5:**
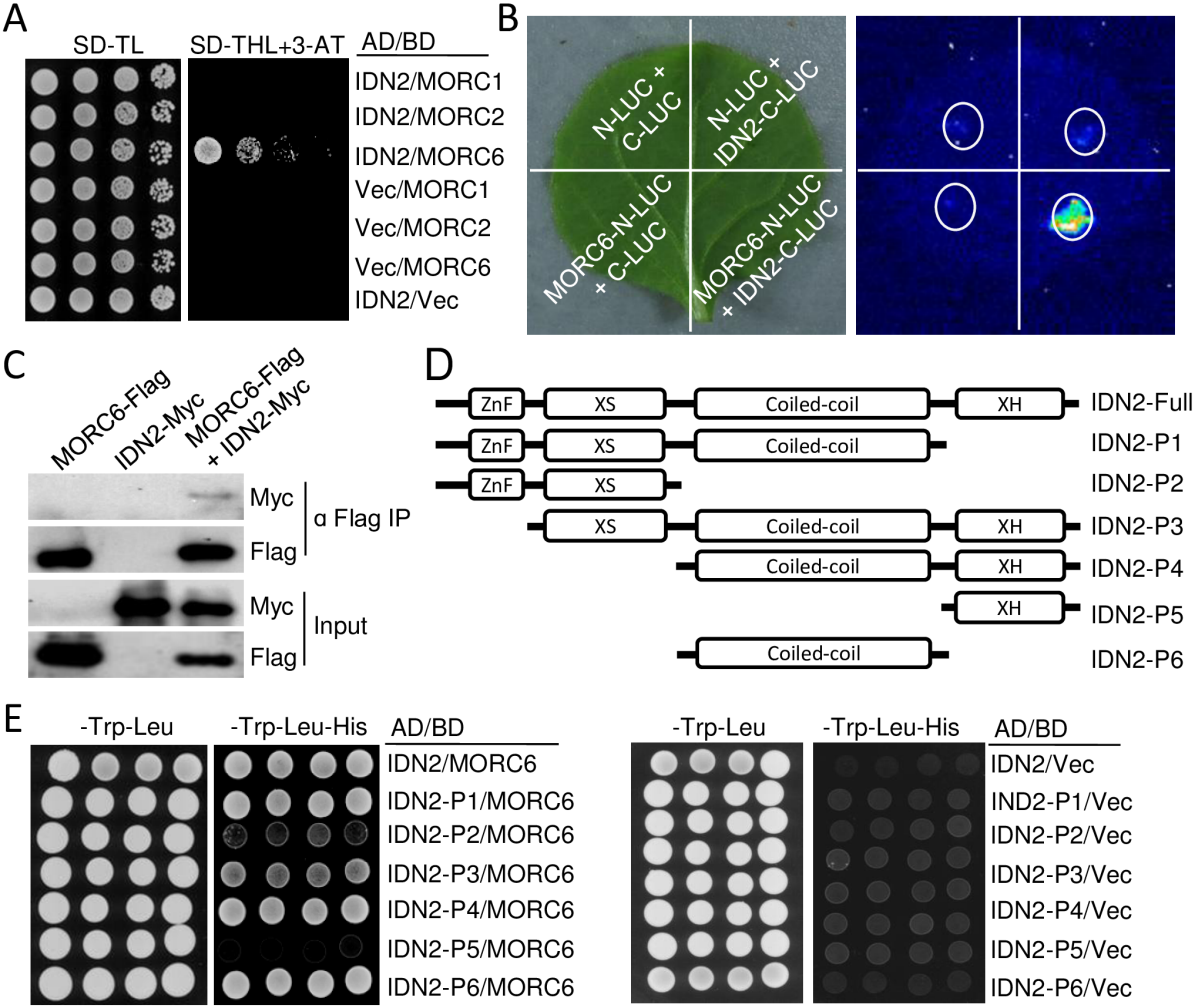
MORC6 interacts with the coiled-coil domain of IDN2. (A) The interaction between MORC6 and IDN2 as determined by a yeast two-hybrid assay. IDN2 was fused with GAL4-AD, whereas MORC1, MORC2, and MORC6 were fused with GAL4-BD. “Vec” represents the empty *GAL4-AD* or *GAL4-BD* vector. A series of diluted strains were grown on two synthetic dropout media: one lacked Trp and Leu (-Trp-Leu), and the other lacked Trp, Leu, and His (-Trp-Leu-His) and was supplemented with 3-AT. (B) The interaction between MORC6 and IDN2 as determined by a split luciferase complementation assay in tobacco (*N*. *benthamiana*). Luciferase activities were determined by luminescence imaging. White circles indicate leaf regions that were infiltrated with the *Agrobacterium* strains containing the indicated constructs. *MORC6* and *IDN2* were fused with *N-LUC* and *C-LUC*, respectively. The empty *N-LUC* and *C-LUC* constructs were transformed as negative controls. (C) The interaction between MORC6 and IDN2 as indicated by co-IP. Transgenic plants harboring *MORC6-Flag* and *IDN2-Myc* transgenes were used in the co-IP experiment. (D) Diagrams indicating full-length and truncated forms of IDN2 that were used in yeast two-hybrid assays. (E) Identification of the IDN2 domain required for interaction with MORC6 as indicated by yeast two-hybrid assays. The full-length and truncated forms of *IDN2* were fused with *GAL4-AD*, and the full-length *MORC6* was fused with *GAL4-BD*. “Vec” represents the empty *GAL4-BD* vector. The growth of four individual colonies is shown for each genotype.

To understand how MORC6 contributes to transcriptional silencing, we performed yeast two-hybrid (Y2H) assay to determine whether MORC6 interacts with any components of the RdDM pathway. The Y2H assay indicated that MORC6 interacts with IDN2 (Fig 5A). The interaction was confirmed by a split luciferase complementation assay in tobacco (Fig 5B). Moreover, we generated transgenic *Arabidopsis* plants harboring *MORC6-Flag* and *IDN2-Myc* transgenes. Using the transgenic plants, we confirmed the interaction between MORC6 and IDN2 by co-immunoprecipitation (co-IP) (Fig 5C). IDN2 is an RdDM component [23,60], which contains four conserved domains: zinc finger domain, XS domain, coiled-coil domain, and XH domain (Fig 5D). The XS domain is necessary for binding to double-stranded RNAs and is required for RdDM [23]. The coiled-coil domain is required for IDN2 dimerization [24,26]. The XH domain is responsible for association with the IDN2 paralogs IDP1 andDP2, facilitating the formation of an IDN2-IDP1/2 complex in the RdDM pathway [24–26]. To determine which domain in IDN2 is required for interaction with MORC6, we generated a series of truncated IDN2 constructs for Y2H assay (Fig 5D). We found that all of the truncated IDN2 fragments containing the coiled-coil domain (P1, P3, P4, and P6) interact with MORC6, whereas the truncated IDN2 fragments without the coiled-coil domain (P2 and P5) fail to interact with MORC6 (Fig 5E), suggesting that the coiled-coil domain is sufficient for the interaction of IDN2 with MORC6. The interaction of MORC6 with IDN2 supports the notion that IDN2 not only acts as a component in the RdDM pathway but also plays a role in transcriptional silencing through a MORC6-dependent pathway.

### MORC6 and SUVH9 interact with SWI3B, SWI3C, and SWI3D

**Fig 6:**
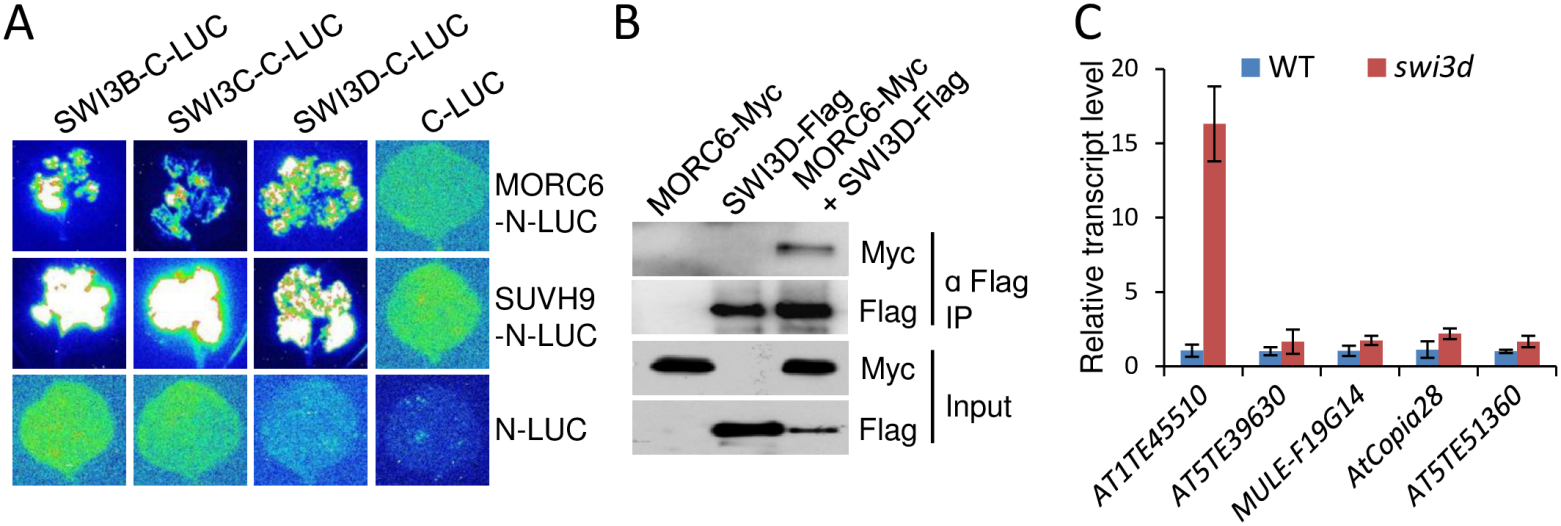
MORC6 and SUVH9 interact with the SWI/SNF chromatin-remodeling complex components SWI3B, SWI3C, and SWI3D. (A) The interaction of MORC6 and SUVH9 with SWI3B, SWI3C, and SWI3D as indicated by split luciferase complementation assays in tobacco. *MORC6* and *SUVH9* were fused with *N-LUC*. *SWI3B*, *SWI3C*, and *SWI3D* were fused with *C-LUC*. The empty *N-LUC* and *C-LUC* constructs were used as negative controls. The indicated constructs were transformed into *Agrobacterium* and were infiltrated into tobacco leaves. (B) Analysis of the interaction between MORC6 and SWI3D by co-IP. Transgenic plants harboring *MORC6-Myc* and *SWI3D-Flag* transgenes were generated and used in the co-IP experiment. (C) The transcript levels of the representative SUVH2/9 and MORC6 common target TEs in the wild type and the *swi3d* mutant as determined by quantitative RT-PCR. Errors are standard deviation of three biological replicates.

IDN2 binds to Pol V-produced noncoding RNAs and physically interacts with SWI3B, a subunit of the SWI/SNF chromatin-remodeling complex, thus contributing to noncoding RNA-mediated transcriptional silencing [52]. The interaction between MORC6 and IDN2 motivated us to determine whether MORC6 interacts with SWI3B and its homologs. Our split luciferase complementation experiment demonstrated that MORC6 interacts not only with SWI3B but also with its homologs SWI3C and SWI3D (Fig 6A). Furthermore, we generated transgenic plants harboring *MORC6-Myc* and *SWI3D-Flag* transgenes and performed co-IP to determine whether MORC6 interacts with SWI3D, demonstrating the interaction between MORC6 and SWI3D (Fig 6B). These results suggest that MORC6 interacts not only with IDN2 but also with the SWI/SNF chromatin-remodeling complex.

Considering MORC6 interacts not only with SUVH9 but also with the SWI/SNF chromatin-remodeling complex, we predicted that SUVH9 may also interact with the SWI/SNF chromatin-remodeling complex. We performed split luciferase complementation assays and found that SUVH9 interacts with the SWI/SNF chromatin-remodeling complex components SWI3B, SWI3C, and SWI3D (Fig 6A). SUVH9 functions redundantly with SUVH2 to bind to methylated DNA and then mediate the occupancy of Pol V on chromatin in the RdDM pathway [38,39]. Although MORC6 interacts with SUVH9, the involvement of MORC6 in transcriptional silencing has been demonstrated to be independent of the function of MORC6 in DNA methylation (Figs 2A–2C, 3A–3D, S6, S7 and S8; S6 and S7 Tables). MORC6 is perhaps linked to methylated DNA by associating with SUVH9 and then interacts with the SWI/SNF chromatin-remodeling complex so as to mediate heterochromatin condensation and to thereby reinforce transcriptional silencing at a downstream step of DNA methylation.

To determine whether the SWI/SNF complex is necessary for the silencing of MORC6 target loci, we carried out quantitative RT-PCR analysis to test the transcript levels of MORC6 target loci in the *swi3d* mutant. The result indicated that in the *swi3d* mutant, the silencing is markedly released in *AT1TE45510* and is either not released or slightly released in the other MORC6 target loci (Fig 6C). We have found that MORC6 and SUVH9 interact not only with SWI3D but also with SWI3B and SWI3C (Fig 6A and 6B), which supports the notion that SWI3B and SWI3C function redundantly with SWI3D to mediate transcriptional silencing at MORC6 target loci.

## Discussion

MORC6/DMS11 was initially identified by two independent groups [40,41]. The study from one group indicated that MORC6 is dispensable for DNA methylation and contributes to transcriptional silencing through heterochromatin condensation [40,44], whereas the study from the other group suggested that MORC6/DMS11 acts as an RdDM component and contributes to Pol V transcription, DNA methylation, and histone H3K9 dimethylation [41]. Our results indicate that MORC6 contributes to DNA methylation at a small number of RdDM target loci (Fig 1A–1E; S1, S2 and S3 Tables), even though the function of MORC6 in DNA methylation is dispensable for transcriptional silencing at MORC6 target loci (Figs 2A–2C, 3A–3D, S6, S7 and S8; S6 and S7 Tables). Moreover, we demonstrate that MORC6 functions in transcriptional silencing at loci targeted by the components of the RdDM pathway (Figs 4A, 4B, S9, S10 and S11; S6 and S7 Tables). We compare the whole-genome DNA methylation data with the RNA-seq data and demonstrate that the release of transcriptional silencing in *morc6* is not associated with loss of DNA methylation (Figs 2A–2C, 3A–3D, S6, S7 and S8; S6 and S7 Tables). The results suggest that the involvement of MORC6 in transcriptional silencing may be caused by its contribution to heterochromatin condensation or H3K9 dimethylation as previously reported [40, 41]. However, how MORC6 cooperates with the RdDM pathway to mediate transcriptional silencing is elusive.

The SRA- and SET-domain-containing proteins SUVH2 and SUVH9 are responsible for binding to methylated DNA and thereby recruiting Pol V to methylated chromatin regions in the RdDM pathway [38,39]. Our RNA-seq data identified many TEs that are either co-up-regulated in *suvh2/9* and *morc6* or specifically up-regulated in *suvh2/9*. We found that in *suvh2/9*, the release of silencing is correlated with DNA hypomethylation in *suvh2/9*-specific up-regulated TEs but not in TEs that are co-up-regulated in *suvh2/9* and *morc6* (Figs 3A–3D, S6, S7 and S8; S6 Table). The results suggest that SUVH2/9 is involved in transcriptional silencing not only by the RdDM pathway but also by a MORC6-dependent pathway. Our genetic analysis demonstrates that SUVH2/9 and MORC6 function in the same pathway at a subset of MORC6 target loci and function at least partially additively at some other MORC6 target loci (Fig 2C), demonstrating that SUVH2/9 and MORC6 cooperate to mediate transcriptional silencing by a mechanism that is distinct from the RdDM pathway. We found that SUVH9 interacts not only with MORC6 but also with the SWI/SNF complex components SWI3B/C/D, suggesting that MORC6 and the SWI/SNF chromatin-remodeling complex are probably recruited to methylated chromatin regions by SUVH2/9, thereby mediating heterochromatin condensation.

To determine whether core components of the RdDM pathway are required for the MORC6-dependent transcriptional silencing, we carried out DNA methylation analysis in *nrpd1*, *rdr2*, *nrpe1*, *drm1/2*, and *idn2*, and found that while DNA methylation tends to be reduced in the RdDM mutants in MORC6 target loci, the reductions of DNA methylation are weaker in MORC6 target loci than in SUVH2/9-specific target loci (Figs 4A, S9, S10, S11, S12, S13 and S14; S6 and S7 Tables). The RdDM components may be involved in the silencing of MORC6 target loci not only through the RdDM pathway but also through the MORC6-dependent pathway. Our previous studies indicated that the RdDM components DTF1/SHH1 and SUVR2 are involved in transcriptional silencing independently of changes in DNA methylation [53,61]. This phenomenon may be explained by the role of the RdDM components in MORC6-dependent transcriptional silencing. The Pol IV largest subunit NRPD1 is responsible for generating 24-nt siRNAs throughout the whole genome, whereas the Pol V largest subunit NRPE1 affects the accumulation of Pol IV-dependent 24-nt siRNAs at RdDM targets that are dispersed in chromosome arms [56,57]. We found that Pol IV-dependent and Pol V-independent 24-nt siRNAs are highly enriched in MORC6 target TEs (Fig 2E; S6 Table), which is consistent with the previous study reporting that MORC6 targets are predominantly present in pericentromeric heterochromatin regions [40]. These results suggest that Pol IV-dependent 24-nt siRNAs may facilitate transcriptional silencing at MORC6 target loci.

Our DNA methylation analyses indicated that the effect of *idn2* on DNA methylation of MORC6 target loci is clearly weaker than the effect of the other canonical RdDM mutations (Figs 4A, 4B, S9, S10, S11, S12, S13 and S14; S6 and S7 Tables). Specifically, we found that in *idn2*, the release of silencing in the SUVH2/9 and MORC6 common target TEs *AT2TE18240* and *AT5TE39630* is not associated with DNA hypomethylation (Fig 4B; S6 Table). These results suggest that IDN2 not only acts as a component in the RdDM pathway but also plays a role in MORC6-dependent transcriptional silencing. In the RdDM pathway, IDN2 binds to Pol V-produced noncoding RNAs and facilitates the function of DRM2 in DNA methylation [27]. Moreover, IDN2 and Pol V-produced noncoding RNAs have been reported to mediate the recruitment of the SWI/SNF chromatin-remodeling complex to chromatin and then lead to nucleosome positioning in a previously study [52]. We demonstrate that MORC6 interacts with IDN2 and the SWI/SNF chromatin-remodeling complex components SWI3B/C/D (Figs 4A–4E, 5A and 5B). The interaction of MORC6 with IDN2 and SWI3B/C/D suggests that the involvement of MORC6 in heterochromatin condensation may depend on IDN2 and the SWI/SNF chromatin-remodeling complex. The involvement of Pol V in heterochromatin condensation has been demonstrated [62]. It is possible that Pol V-produced noncoding RNAs are required not only for the RdDM pathway but also for the MORC6-dependent heterochromatin condensation.

**Fig 7:**
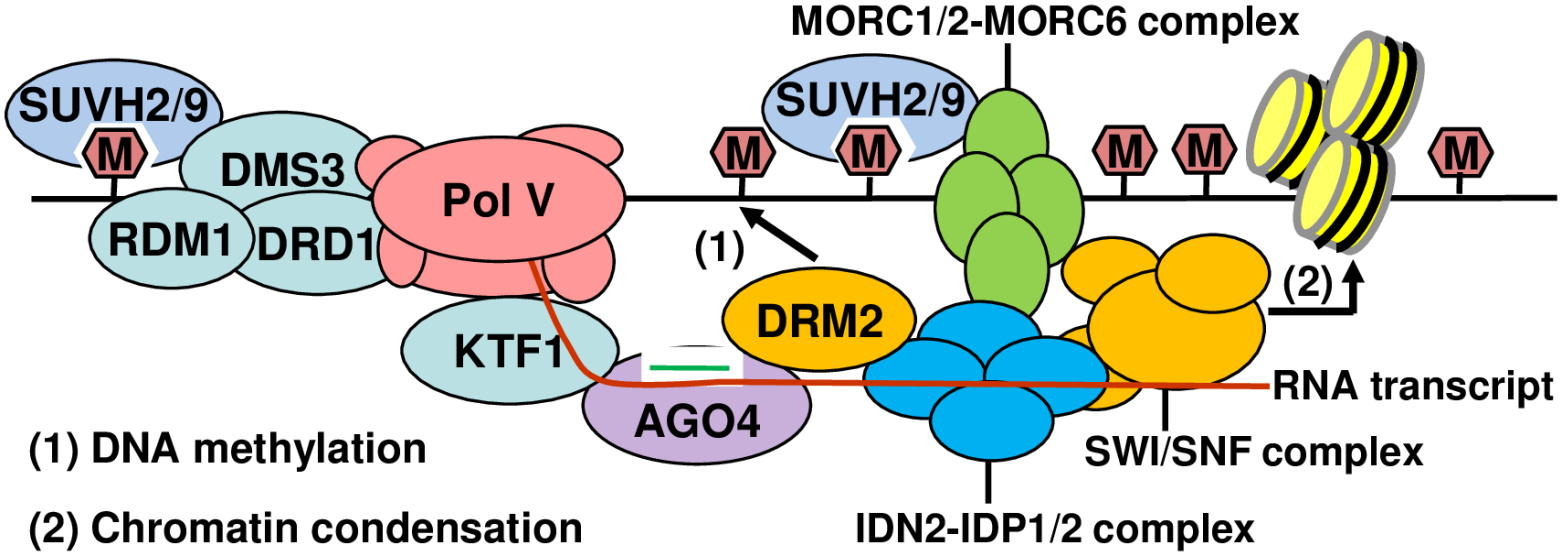
Model for the roles of SUVH2/9 in RNA-directed DNA methylation and chromatin condensation in *Arabidopsis*. The methylated-DNA-binding proteins SUVH2/9 interact with the DDR complex to recruit Pol V to chromatin and thereby facilitate a self-reinforcing loop between Pol V transcription and DNA methylation in the RNA-directed DNA methylation pathway. Moreover, SUVH2/9 interact with the MORC1/2-MORC6 complex, which then interacts with IDN2 and the SWI/SNF complex to mediate chromatin condensation. IDN2 is required not only for RNA-directed DNA methylation but also for chromatin condensation. The physical interaction between SUVH2/9, MORCs, IDN2, and the SWI/SNF complex components SWI3/C/D facilitates the interplay between DNA methylation and chromatin condensation, thus enhancing transcriptional silencing at their common target loci.

Together, our study suggests that MORC6 interacts with SUVH2/9, IDN2, and the SWI/SNF complex SWI3B/C/D and may either act at a downstream step of DNA methylation or facilitate a self-reinforcing loop between DNA methylation and chromatin condensation. In the RdDM pathway, the binding of SUVH2/9 to methylated DNA facilitates the recruitment of Pol V to chromatin and thereby mediates DNA methylation [38,39]. The interaction between SUVH2/9 and MORC6 suggest that the binding of SUVH2/9 to methylated DNA may be also required for the recruitment of MORC6 to chromatin. Thereafter, MORC6 interacts with IDN2 and then with the SWI/SNF complex to mediate heterochromatin condensation (Fig 7). These components work together to mediate the link between DNA methylation and heterochromatin condensation, thus enhancing transcriptional silencing at methylated genomic regions.

## Materials and Methods

### Plant materials, cloning, and growth conditions

*Arabidopsis* plant material was grown on Murashige and Skoog (MS) medium at 22°C with 16-h light/8-h dark (long-day conditions). When adult plants or flowers were needed, 2-week-old seedlings were transplanted from MS plates into soil and grown in a growth room under long-day conditions at 22°C. The *Arabidopsis* T-DNA lines included *nrpd1a-3* (SALK_128428), *rdr2-1* (CS66076), *nrpe1-11* (SALK_029919C), *suvh2* (Gabi_kat_516A07), *suvh9* (SALK_048033), *suvh2suvh9* [55], *idn2-5* (FLAG_550B05), *drm1/2* (CS16383), *swi3d* (SALK_100310), *morc6* (GK-599B06-023140), and *morc2* (SALK_021267C). The *morc1* mutation was introduced into the *morc2morc6* double mutant by CRISPR-CAS9 to obtain the *morc1morc2morc6* triple mutant. The *Flag*- and/or *Myc*-tagged *IDN2*, *MORC6*, and *SUVH9* transgenic plants were obtained as previously described [24,39]. The full-length *SWI3D* genomic sequence was fused with the *Flag* tag at its C-terminus and cloned into the modified plant expression vector *pCAMBIA1305* for transformation.

### Analyses of DNA methylation

Genomic DNA was extracted from 2-week-old seedlings by CTAB and purified with phenol:chloroform (1:1). For PCR-based DNA methylation analysis, 100 ng of genomic DNA was cleaved by HaeIII, Alu I, or McrBC, followed by amplification of the digested DNA at the indicated loci. HaeIII and AluI are sensitive to methylated DNA, and McrBC specifically cleaves methylated DNA. For whole-genome bisulfite sequencing, genomic DNA was extracted from 2-week-old seedlings and subjected to sodium bisulfite conversion (EpiTect Bisulfite Kits, Qiagen). Libraries were paired-end sequenced on Illumina HiSeq 2000 (BGI, China). For whole-genome DNA methylation analysis, reads were mapped to the TAIR10 *Arabidopsis* genome by Bismark (v0.10.0), allowing up to two mismatches. The methylation level of each cytosine site was represented by the percentage of the number of reads reporting a C relative to the total number of reads reporting a C or T. Only sites with at least five-fold coverage were included in the results. DNA methylation was calculated in every 50-bp window and was compared in the wild type and mutants. Windows with absolute DNA methylation difference of 10% and p<0.01 (Fisher’s exact test) were selected. We combined proximal differentially methylated windows to generate differentially methylated regions (DMRs) when the gaps were no more than 50 bp. Two DMRs were considered overlapping when the overlap exceeded 50 bp. The methylation levels of genes and TEs were estimated by pooling the read counts that show at least five-fold coverage. DNA methylation at CG, CHG, and CHH sites was separately evaluated. To evaluate the correlation between expression and DNA methylation, we determined the DNA methylation levels of different classes of differentially expressed loci.

### RNA sequencing-based gene expression assay

Total RNA was extracted from 2-week-old seedlings and used for library construction and Illumina sequencing. For library preparation, RNA was purified from total RNA using poly-T oligo-attached magnetic beads. The libraries were generated using the NEBNext Ultra RNA Library Prep Kit for Illumina (NEB, USA) and subjected to deep sequencing on an Illumina Hiseq 2500 platform. For data analysis, the adaptor sequences were removed, and reads were then mapped to the *Arabidopsis* genome (TAIR10, http://www.arabidopsis.org/) using the TopHat v2.0.6 program [63] and allowing a maximum of two mismatches. Gene and TE expression was analyzed with the Cufflinks (v2.0.1) package. Expression change was evaluated by combining fold-change of normalized reads (fold-change> 2) and Fisher's exact test (p < 0.01). Heat maps of differentially expressed genes were generated by the Gplots package in R.

### Small RNA deep sequencing

The small RNA data of the wild type, *nrpd1*, and *nrpe1* were produced in our previous study [39]. Raw small RNA reads were processed to remove adapter sequences and were then mapped to SUVH2/9 and MORC6 target TEs and genes with the Bowtie program. TE body sequences and 1-Kb gene promoter sequences targeted by SUVH2/9 and MORC6 were used for analysis. In each sequence, the reads of 24-nt siRNAs were counted and were then normalized by the total reads of the small RNA library. Reads per ten million (RPTM) are indicated for SUVH2/9 and MORC6 target TEs and genes in the wild type, *nrpd1*, and *nrpe1*.

### Analyses of RNA transcripts by RT-PCR

Total RNA was extracted by TRI Reagent (T9424, Sigma) as described previously [28]. For RT-PCR, the ReverTra Ace qPCR RT Master Mix with the gDNA remover kit was used for reverse transcription, and RNA transcript levels were determined by RT-PCR (FSQ301, Toyobo). The quantitative RT-PCR experiment was biologically repeated, and the results of three biological replicates are shown. The primers used for RT-PCR are listed in S8 Table.

### Yeast two-hybrid assay

The yeast two-hybrid assay was performed by yeast mating between the strains Y187 and AH109. Sequences of cDNAs were fused with *GAL4-AD* and *GAL4-BD* in the *pGADT7* and *pGBKT7* vectors, respectively. The *pGADT7* constructs were transformed into the yeast strain Y187 and grown on the synthetic dropout medium minus Leu (SD-Leu). The *pGBKT7* constructs were transformed into the yeast strain AH109 and grown on the SD medium minus Trp (SD-Trp). For yeast mating, the two types of positive yeast strains were mixed and incubated in 100 μL of YPD on a shaker overnight. The mixture was streaked on the synthetic dropout medium minus Trp and Leu (SD-Trp-Leu) to obtain positive mating strains. The synthetic dropout medium minus Trp, Leu, and His (SD-Trp-Leu-His) that contains 5 mM 3-AT was used for screening the positive yeast strains in which the GAL-AD fusion protein interacts with the GAL4-BD fusion protein. To confirm the interactions, the *pGADT7* and *pGBKT7* constructs were co-transformed into the yeast strain AH109. The positive yeast strains were selected from the SD-Trp-Leu medium and then grown on the SD-Trp-Leu-His medium supplemented with 5 mM 3-AT. The primers used for cloning are listed in S8 Table.

### Split luciferase complementation assay

Full-length cDNAs were fused with *N-LUC* or *C-LUC* at their C-termini in the *pCAMBIA1300* vector and transformed into the *Agrobacterium tumefaciens GV3101*. The positive strains were selected and then cultured in YEB medium. When the OD_600_ value of the cultures reached 0.6, the cultures were resuspended in a solution containing 10 mM MgCl_2_, 10 mM MES(pH 5.7), and 100μM acetosyringone, and then incubated for 4 h at 28°C. The strains containing *N-LUC* and *C-LUC* fusion constructs were mixed in equal volumes and infiltrated into the leaves of tobacco (*Nicotiana benthamiana*) with a syringe. The luminescence images were captured after 48 h. The DNA primers used for the *N-LUC* and *C-LUC* fusion construction are listed in S8 Table.

### Co-immunoprecipitation assay

To detect the interaction between MORC6 and IDN2 or SWI3D, we crossed *MORC6-Flag* and *MORC6-Myc* transgenic plants to *IDN2-Myc* and *SWI3D-Flag* transgenic plants, respectively. The F_1_ hybrids and their parental plants were used for co-IP analysis. A 1-g quantity of 2-week-old seedlings was ground and homogenized in 5 ml of lysis buffer. After the cell debris was removed by centrifugation, the supernatant was incubated with Anti-Flag M2 Affinity Gel (Sigma, A2220) in lysis buffer at 4°C for 2.5 h. The resins were precipitated and washed four times with lysis buffer. The agarose-bound proteins were eluted with 3xFlag peptide and run on an SDS-PAGE gel for western blotting.

### Accession number

The whole-genome bisulfite sequencing and RNA sequencing data have been deposited in the Gene Expression Omnibus (GEO) database (accession no. GSE80370).

## Supporting Information

S1 Fig Compositions of genomic locations of hypo-DMRs identified in the *suvh2/9* and *morc6* mutants. Genomic locations were classified into genes, TEs, and other uncharacterized regions. Percentages of all types of genomic locations are shown.
(TIF)

S2 Fig Display of DNA methylation of selected hypo-DMRs in the wild type, *suvh2/9*, and *morc6* as determined by the whole-genome bisulfite sequencing data. (A) DNA methylation of selected overlapping hypo-DMRs in *suvh2/9* and *morc6* as determined by bisulfite sequencing. These hypo-DMRs (Class I) are shown in Fig 1E. (B) DNA methylation of selected *suvh2/9*-specific hypo-DMRs. These hypo-DMRs (Class II) are shown in Fig 1E. The percentage of cytosine methylation in the cytosine contexts CG, CG, and CHH is represented by blue, red, and green lines, respectively.
(TIF)

S3 Fig Generation of the *morc1* mutation by the CRISPR-CAS9 system. The synthetic-guide RNA (sgRNA) was designed in the 5^th^ exon of *MORC1*. The target sequence of the sgRNA was highlighted in green. The target site precedes a TGG (in orange), which is a protospacer adjacent motif (PAM) required for the function of CRISPR/CAS9 system. In the *morc1* mutant, a cytosine insertion indicated in purple leads to premature termination of transcription.
(TIF)

S4 Fig Determination of the DNA methylation levels of the RdDM target loci *solo LTR*, *AtSN1*, and *ETR7* in *morc6* and *morc1/2/6*. **G**enomic DNA was cleaved by the DNA methylation-sensitive restriction enzyme AluI or HaeIII followed by PCR to determine DNA methylation. The DNA methylation levels of the RdDM target loci *solo LTR*, *AtSN1*, and *ETR7* were determined in the wild type, *nrpe1*, *suvh2/9*, *morc6*, and *morc1/2/6*. *ACT2* was used as a loading control.
(TIF)

S5 Fig DNA methylation of the highly methylated locus *TSI* (*transcriptional silent information*) in *nrpe1*, *suvh2/9*, *morc6*, and *morc1/2/6* relative to the wild type. Genomic DNA was digested by McrBC and then subjected to PCR. McrBC is a restriction enzyme that specifically works on methylated DNA. Genomic DNA without McrBC digestion was amplified as a control.
(TIF)

S6 Fig Box plots showing DNA methylation levels of up-regulated TEs and genes in *morc6* and *suvh2/9*. Up-regulated TEs and genes were selected for box plotting when significantly methylated (>20% methylation for TEs; >5% methylation for genes) in the wild type. Asterisks indicate statistical significance (t-test; * p<0.05, ** p<0.01). p value is shown for each sample.
(TIF)

S7 Fig Effect of *suvh2/9* and *morc6* on DNA methylation of transcriptionally up-regulated TEs and genes identified in *suvh2/9* and *morc6*. (A, C) Scatter plots showing CG and CHG methylation of up-regulated TEs and genes identified in *suvh2/9* and *morc6*. Blue dots, TEs and genes that are specifically up-regulated in *suvh2/9*; Red dots, TEs and genes that are co-up-regulated in *suvh2/9* and *morc6*; Green dots, TEs and genes that are specifically up-regulated in *morc6*. (B, D) Box plots showing CG and CHG methylation of transcriptionally up-regulated TEs in *suvh2/9* and *morc6* relative to the wild type. Asterisks indicate statistical significance (t-test; * p<0.05, ** p<0.01). p value is shown for each sample.
(TIF)

S8 Fig Determination of DNA methylation levels of TEs by locus-specific bisulfite sequencing analysis. DNA methylation of *AT4TE09800*, *AT4TE09845*, and *AT5TE39630* was determined by locus-specific bisulfite sequencing analysis in the wild type, *suvh2/9* and *morc6*. The DNA methylation levels are separately shown in the three types of cytosine contexts: CG, CHG, and CHH. H represents A, T, and C.
(TIF)

S9 Fig Scatter plots showing effect of *nrpd1*, *rdr2*, *nrpe1*, *drm1/2*, and *idn2* on DNA methylation of transcriptionally up-regulated TEs and genes identified in *suvh2/9* and *morc6*. Total C, CG, CHG, and CHH methylation of transcriptionally up-regulated TEs and genes identified in *suvh2/9* and *morc6* are shown. Blue dots represent TEs and genes that are transcriptionally up-regulated in *suvh2/9* but not in *morc6*, whereas red dots represent TEs and genes that are transcriptionally co-up-regulated in *suvh2/9* and *morc6*. DNA methylation of the TEs and genes in *nrpd1*, *rdr2*, *nrpe1*, *drm1/2*, and *idn2* was separately compared to that in the wild type by scatter plots.
(TIF)

S10 Fig Box plots showing effect of *nrpd1*, *rdr2*, *nrpe1*, *drm1/2*, and *idn2* on DNA methylation of transcriptionally up-regulated TEs identified in *suvh2/9* and *morc6*. (A) DNA methylation of TEs that are transcriptionally up-regulated in *suvh2/9* but not in *morc6*. (B) DNA methylation of TEs that are transcriptionally co-up-regulated in *suvh2/9* and *morc6*. DNA methylation was analyzed at either total cytosine sites or the three different cytosine contexts CG, CHG, and CHH. Asterisks indicate statistical significance (t-test; * p<0.05, ** p<0.01). p value is shown for each sample.
(TIF)

S11 Fig Box plots showing effect of *nrpd1*, *rdr2*, *nrpe1*, *drm1/2*, and *idn2* on promoter DNA methylation of transcriptionally up-regulated genes identified in *suvh2/9* and *morc6*. (A) Promoter DNA methylation of genes that are transcriptionally up-regulated in *suvh2/9* but not in *morc6*. (B) Promoter DNA methylation of genes that are transcriptionally co-up-regulated in *suvh2/9* and *morc6*. Genes were included for analysis only when their promoter DNA methylation is higher than 5%. DNA methylation was analyzed at either total cytosine sites or the three different cytosine contexts CG, CHG, and CHH. Asterisks indicate statistical significance (t-test; * p<0.05, ** p<0.01). p value is shown for each sample.
(TIF)

S12 Fig Scatter plots showing effect of the RdDM mutations on DNA methylation of TEs that are specifically up-regulated in *morc6*. Green dots shown in the scatter plots represent total C, CG, CHG, and CHH methylation of *morc6* specific up-regulated TEs in the wild type and the mutants including *nrpd1*, *rdr2*, *nrpe1*, *drm1/2*, and *idn2*.
(TIF)

S13 Fig Scatter plots showing effect of the RdDM mutations on DNA methylation of genes that are specifically up-regulated in *morc6*. Green dots shown in the scatter plots represent total C, CG, CHG, and CHH methylation of *morc6* specific up-regulated genes in the wild type and the mutants including *nrpd1*, *rdr2*, *nrpe1*, *drm1/2*, and *idn2*.
(TIF)

S14 Fig Box plots showing effect of the RdDM mutations on DNA methylation of TEs and genes that are specifically up-regulated in *morc6*. (A) DNA methylation of TEs that are specifically up-regulated in *morc6*. (B) DNA methylation of genes that are specifically up-regulated in *morc6*. Total cytosine, CG, CHG, and CHH methylation are separately indicated. Asterisks indicate statistical significance (t-test; * p<0.05, ** p<0.01). p value is shown for each sample.
(TIF)

S15 Fig RNA transcript levels of the Pol V-produced transcripts *IGN5* and *IGN25*. The RNA transcript levels of *IGN5* and *IGN25* were determined by quantitative RT-PCR in the wild type, *nrpe1*, *suvh2/9*, *morc6*, and *morc1/2/6*. The actin gene *ACT2* was used as an internal control.
(TIF)

S1 Table Summary of DMRs identified in *suvh9*, *suvh2suvh9*, *morc6*, and *suvh9morc6* relative to the wild type. (XLSX)

S2 Table DNA methylation and 24-nt siRNA accumulation of *suvh2/9* hypo-DMRs. (XLSX)

S3 Table DNA methylation and 24-nt siRNA accumulation of *morc6* hypo-DMRs. (XLSX)

S4 Table Differentially expressed TEs in *suvh2/9* and *morc6* relative to the wild type. (XLSX)

S5 Table Differentially expressed genes in *suvh2/9* and *morc6* relative to the wild type. (XLSX)

S6 Table DNA methylation and 24-nt siRNA accumulation at up-regulated TEs in *suvh2/9* and *morc6*. (XLSX)

S7 Table DNA methylation and 24-nt siRNA accumulation at up-regulated genes in *suvh2/9* and *morc6*. (XLSX)

S8 Table List of DNA oligonucleotides used in this study. (XLS)
