# Supplementary figures and images for: Two Components of the RNA-Directed DNA Methylation Pathway Associate with MORC6 and Silence Loci Targeted by MORC6 in Arabidopsis
Source: PLoS Genet. 2016 May 12;12(5):e1006026. doi: 10.1371/journal.pgen.1006026 (PMC4865133; doi:10.1371/journal.pgen.1006026)

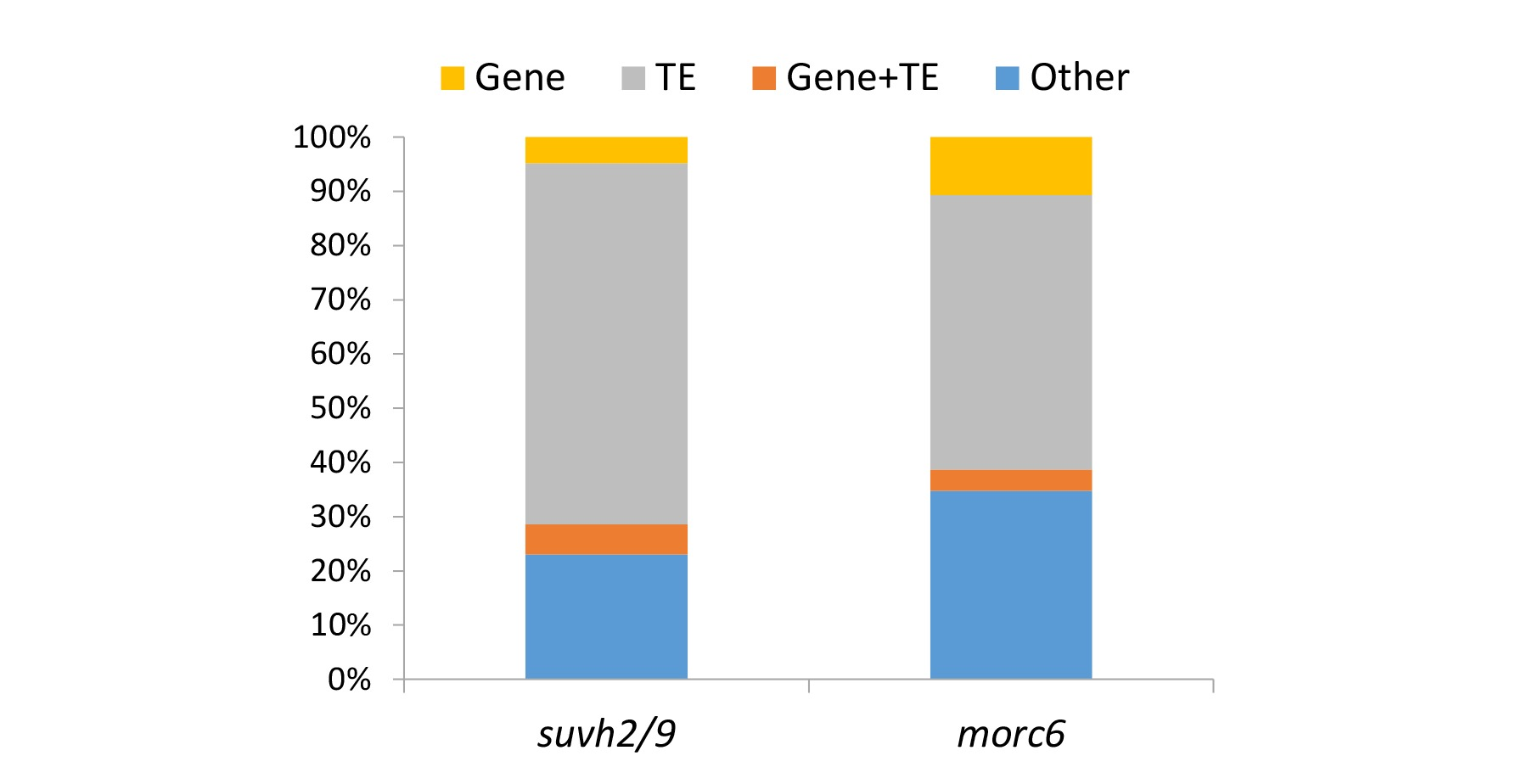

Supplement: S1 Fig — Genomic locations were classified into genes, TEs, and other uncharacterized regions. Percentages of all types of genomic locations are shown. (TIF) [file pgen.1006026.s001.tif]

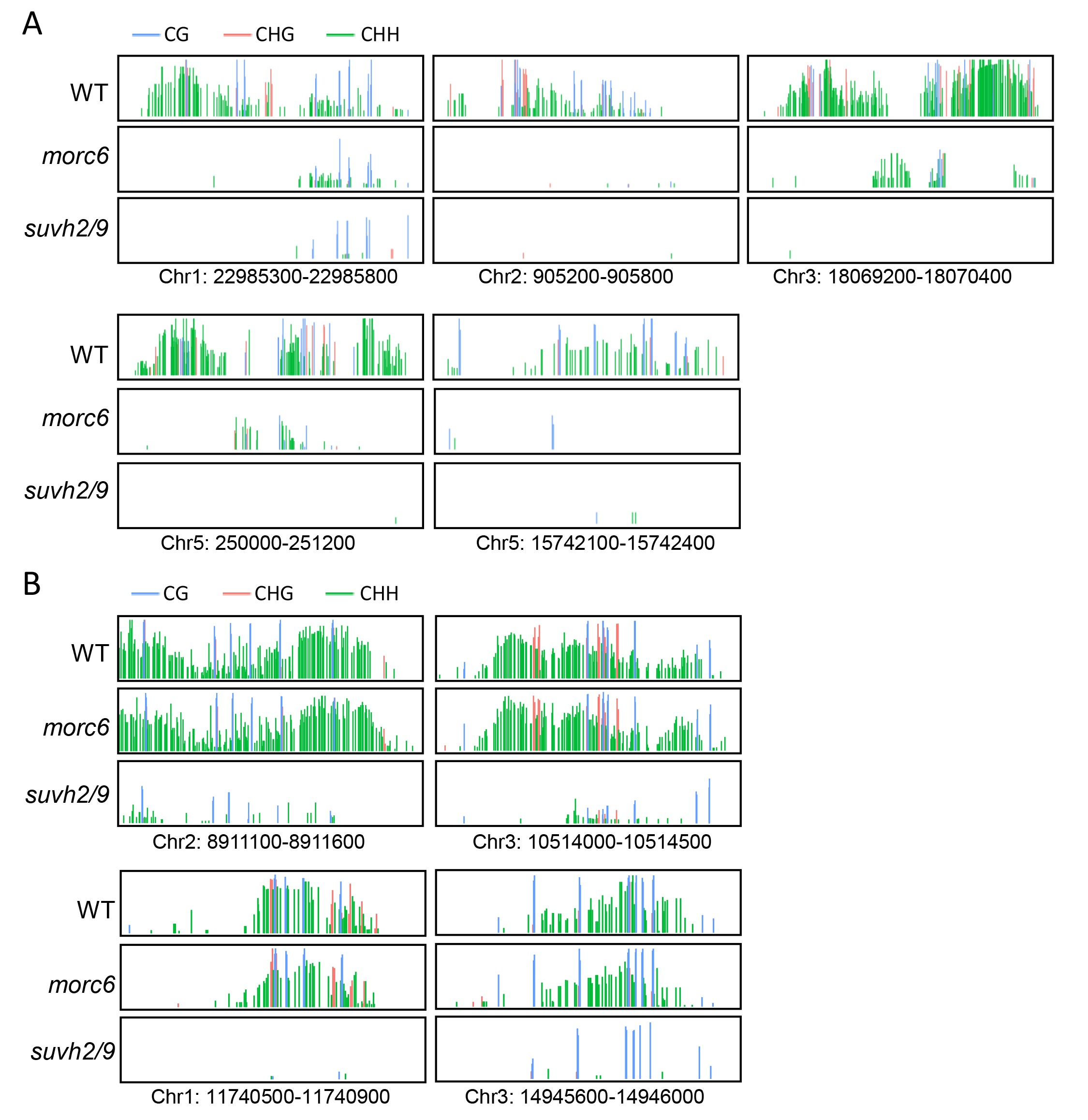

Supplement: S2 Fig — (A) DNA methylation of selected overlapping hypo-DMRs in suvh2/9 and morc6 as determined by bisulfite sequencing. These hypo-DMRs (Class I) are shown in Fig 1E. (B) DNA methylation of selected suvh2/9-specific hypo-DMRs. These hypo-DMRs (Class II) are shown in Fig 1E. The percentage of cytosine methylation in the cytosine contexts CG, CG, and CHH is represented by blue, red, and green lines, respectively. (TIF) [file pgen.1006026.s002.tif]

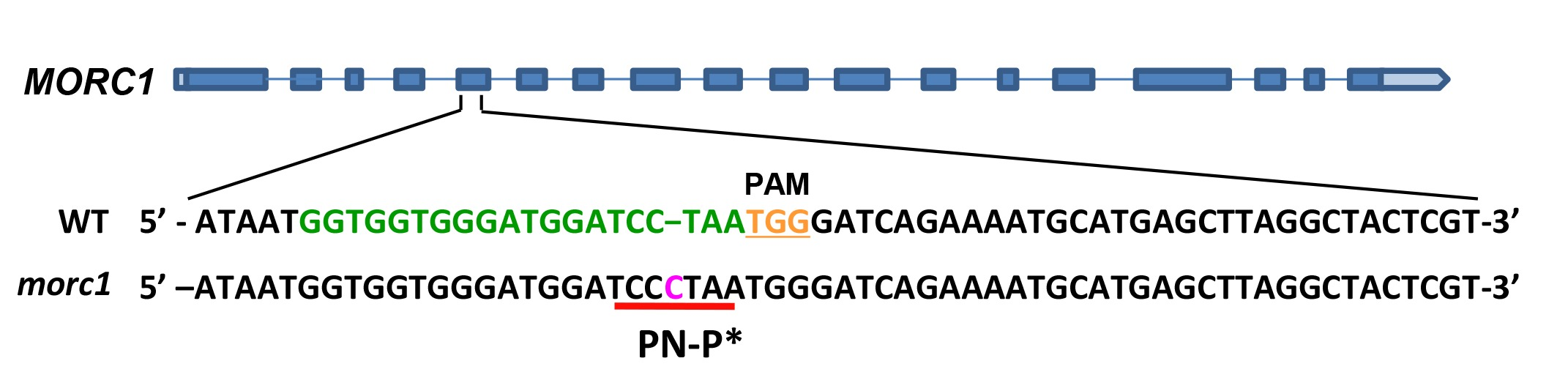

Supplement: S3 Fig — The synthetic-guide RNA (sgRNA) was designed in the 5th exon of MORC1. The target sequence of the sgRNA was highlighted in green. The target site precedes a TGG (in orange), which is a protospacer adjacent motif (PAM) required for the function of CRISPR/CAS9 system. In the morc1 mutant, a cytosine insertion indicated in purple leads to premature termination of transcription. (TIF) [file pgen.1006026.s003.tif]

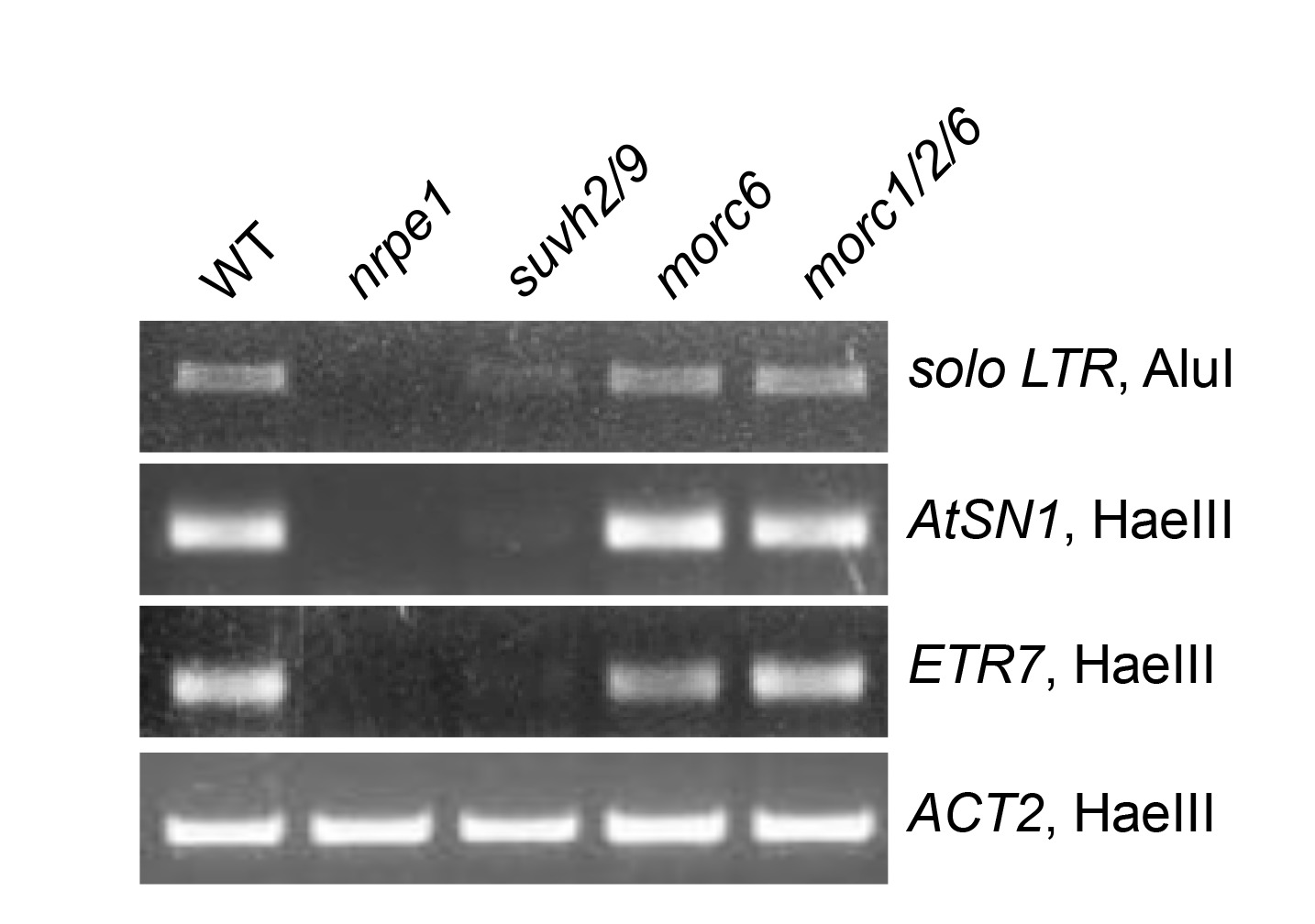

Supplement: S4 Fig — Genomic DNA was cleaved by the DNA methylation-sensitive restriction enzyme AluI or HaeIII followed by PCR to determine DNA methylation. The DNA methylation levels of the RdDM target loci solo LTR, AtSN1, and ETR7 were determined in the wild type, nrpe1, suvh2/9, morc6, and morc1/2/6. ACT2 was used as a loading control. (TIF) [file pgen.1006026.s004.tif]

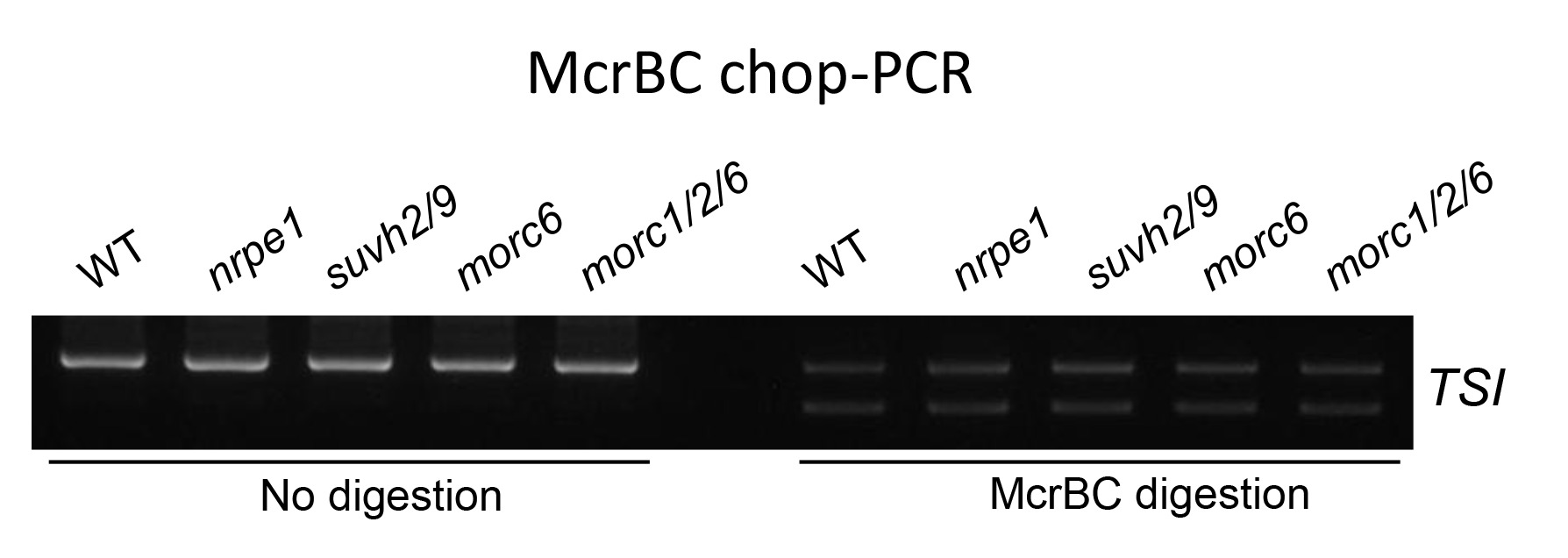

Supplement: S5 Fig — Genomic DNA was digested by McrBC and then subjected to PCR. McrBC is a restriction enzyme that specifically works on methylated DNA. Genomic DNA without McrBC digestion was amplified as a control. (TIF) [file pgen.1006026.s005.tif]

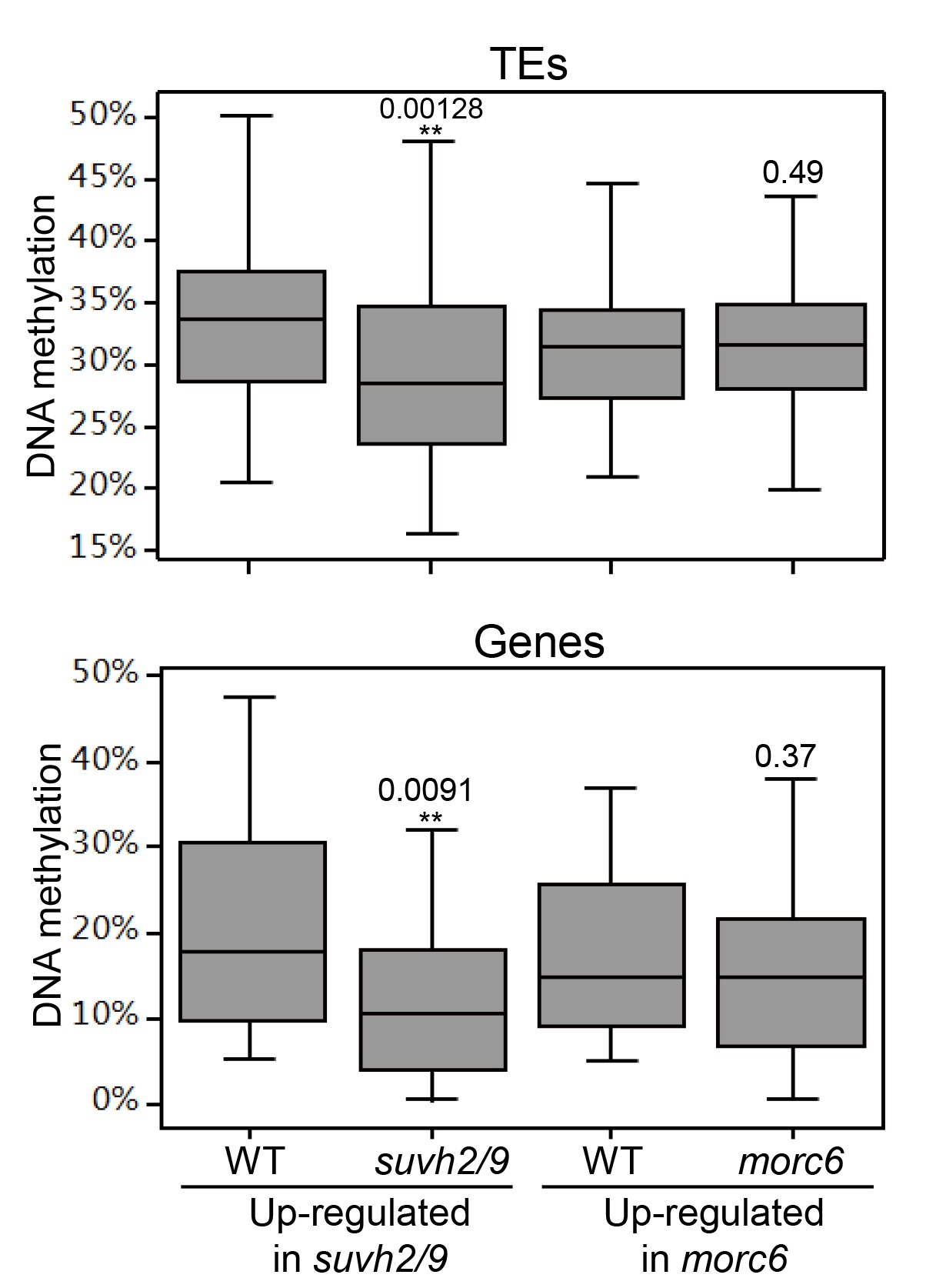

Supplement: S6 Fig — Up-regulated TEs and genes were selected for box plotting when significantly methylated (>20% methylation for TEs; >5% methylation for genes) in the wild type. Asterisks indicate statistical significance (t-test; * p<0.05, ** p<0.01). p value is shown for each sample. (TIF) [file pgen.1006026.s006.tif]

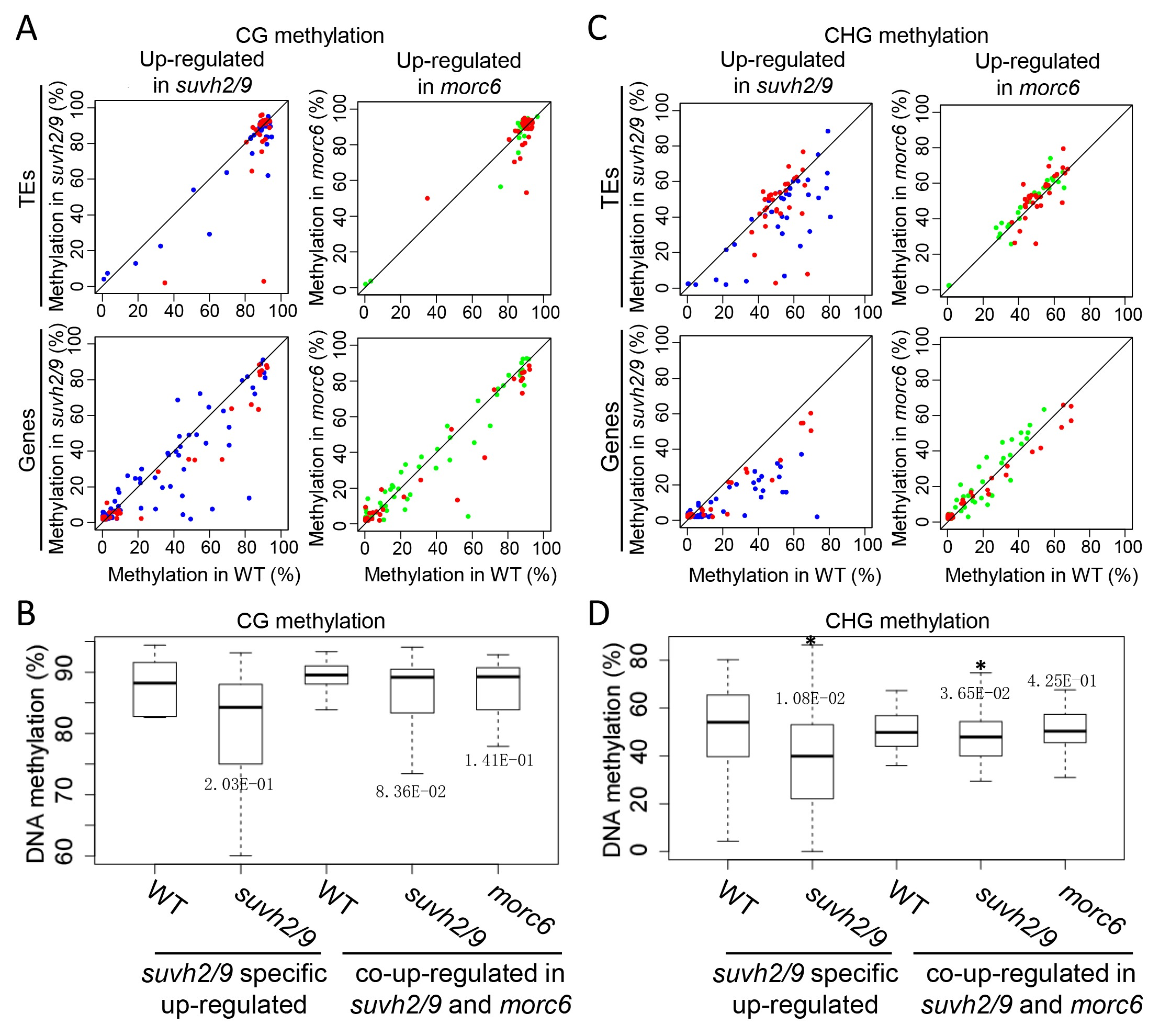

Supplement: S7 Fig — (A, C) Scatter plots showing CG and CHG methylation of up-regulated TEs and genes identified in suvh2/9 and morc6. Blue dots, TEs and genes that are specifically up-regulated in suvh2/9; Red dots, TEs and genes that are co-up-regulated in suvh2/9 and morc6; Green dots, TEs and genes that are specifically up-regulated in morc6. (B, D) Box plots showing CG and CHG methylation of transcriptionally up-regulated TEs in suvh2/9 and morc6 relative to the wild type. Asterisks indicate statistical significance (t-test; * p<0.05, ** p<0.01). p value is shown for each sample. (TIF) [file pgen.1006026.s007.tif]

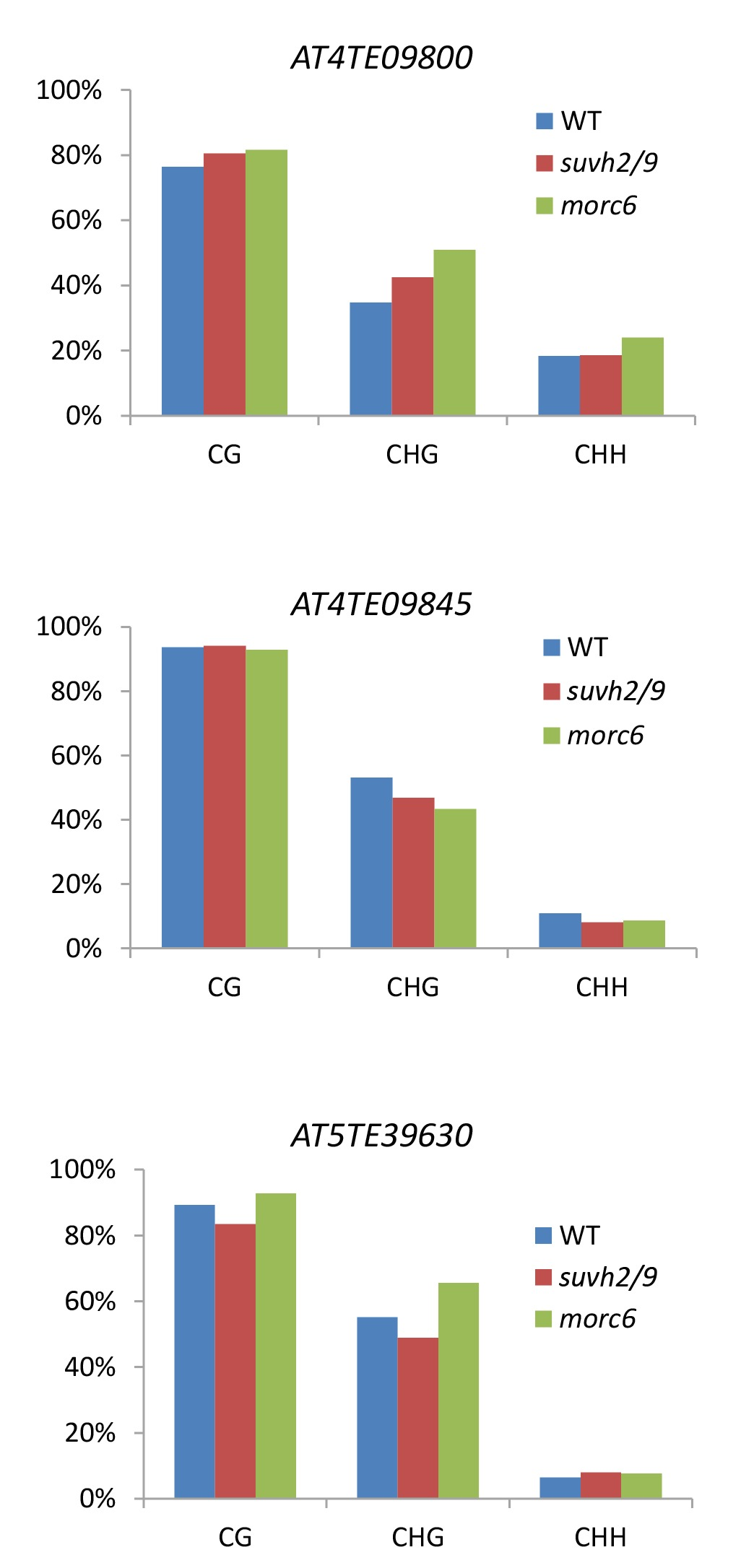

Supplement: S8 Fig — DNA methylation of AT4TE09800, AT4TE09845, and AT5TE39630 was determined by locus-specific bisulfite sequencing analysis in the wild type, suvh2/9 and morc6. The DNA methylation levels are separately shown in the three types of cytosine contexts: CG, CHG, and CHH. H represents A, T, and C. (TIF) [file pgen.1006026.s008.tif]

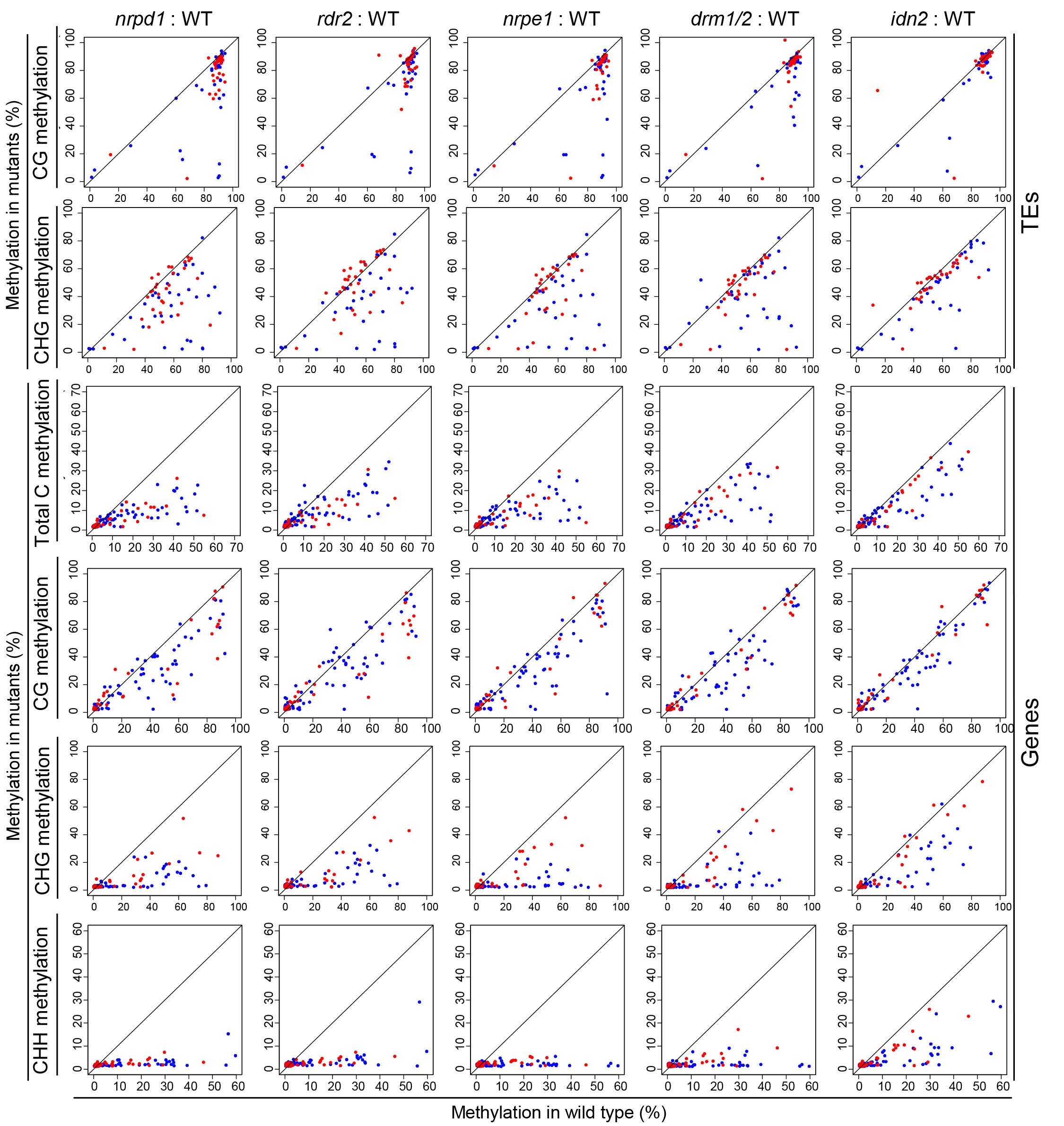

Supplement: S9 Fig — Total C, CG, CHG, and CHH methylation of transcriptionally up-regulated TEs and genes identified in suvh2/9 and morc6 are shown. Blue dots represent TEs and genes that are transcriptionally up-regulated in suvh2/9 but not in morc6, whereas red dots represent TEs and genes that are transcriptionally co-up-regulated in suvh2/9 and morc6. DNA methylation of the TEs and genes in nrpd1, rdr2, nrpe1, drm1/2, and idn2 was separately compared to that in the wild type by scatter plots. (TIF) [file pgen.1006026.s009.tif]

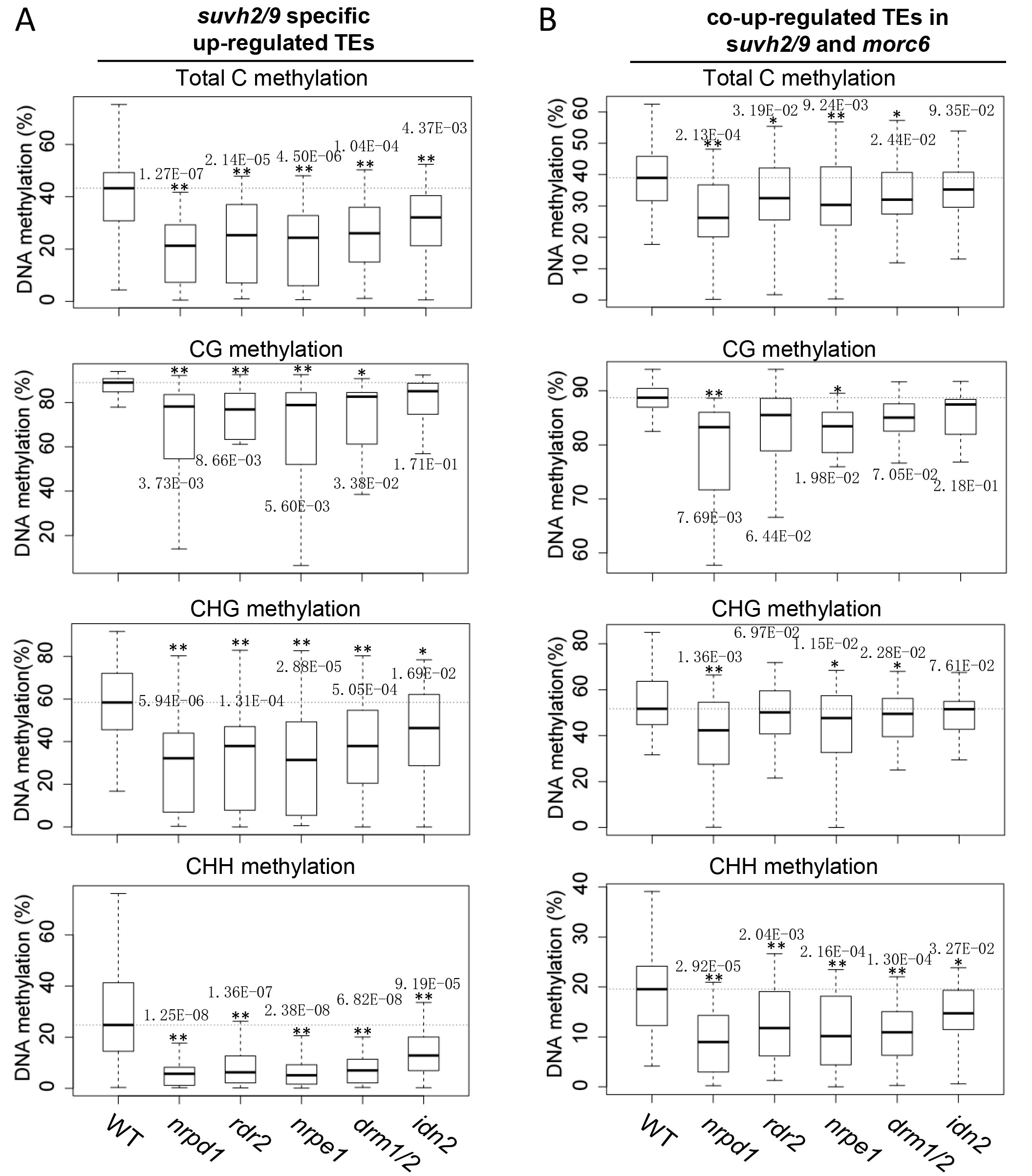

Supplement: S10 Fig — (A) DNA methylation of TEs that are transcriptionally up-regulated in suvh2/9 but not in morc6. (B) DNA methylation of TEs that are transcriptionally co-up-regulated in suvh2/9 and morc6. DNA methylation was analyzed at either total cytosine sites or the three different cytosine contexts CG, CHG, and CHH. Asterisks indicate statistical significance (t-test; * p<0.05, ** p<0.01). p value is shown for each sample. (TIF) [file pgen.1006026.s010.tif]

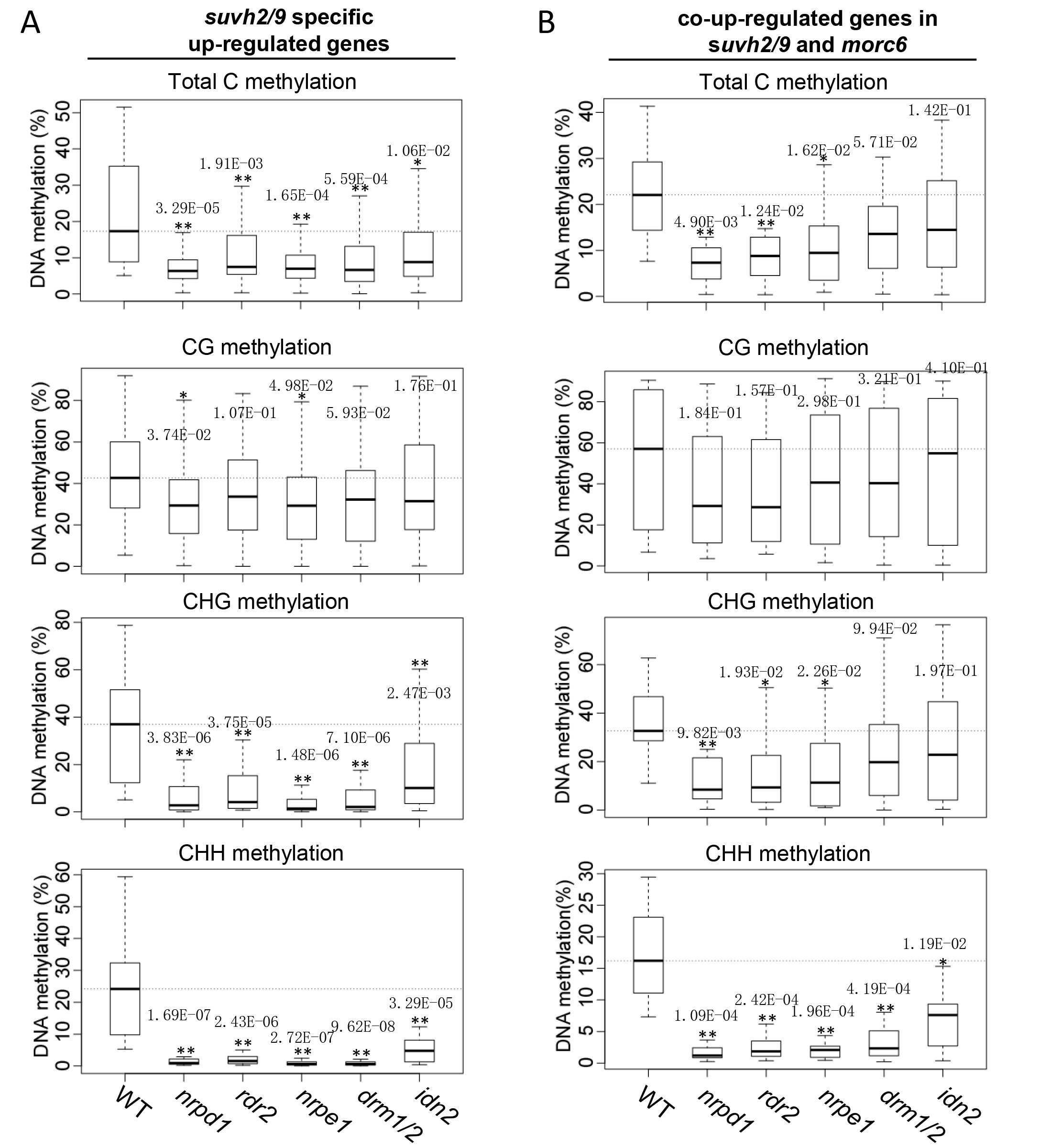

Supplement: S11 Fig — (A) Promoter DNA methylation of genes that are transcriptionally up-regulated in suvh2/9 but not in morc6. (B) Promoter DNA methylation of genes that are transcriptionally co-up-regulated in suvh2/9 and morc6. Genes were included for analysis only when their promoter DNA methylation is higher than 5%. DNA methylation was analyzed at either total cytosine sites or the three different cytosine contexts CG, CHG, and CHH. Asterisks indicate statistical significance (t-test; * p<0.05, ** p<0.01). p value is shown for each sample. (TIF) [file pgen.1006026.s011.tif]

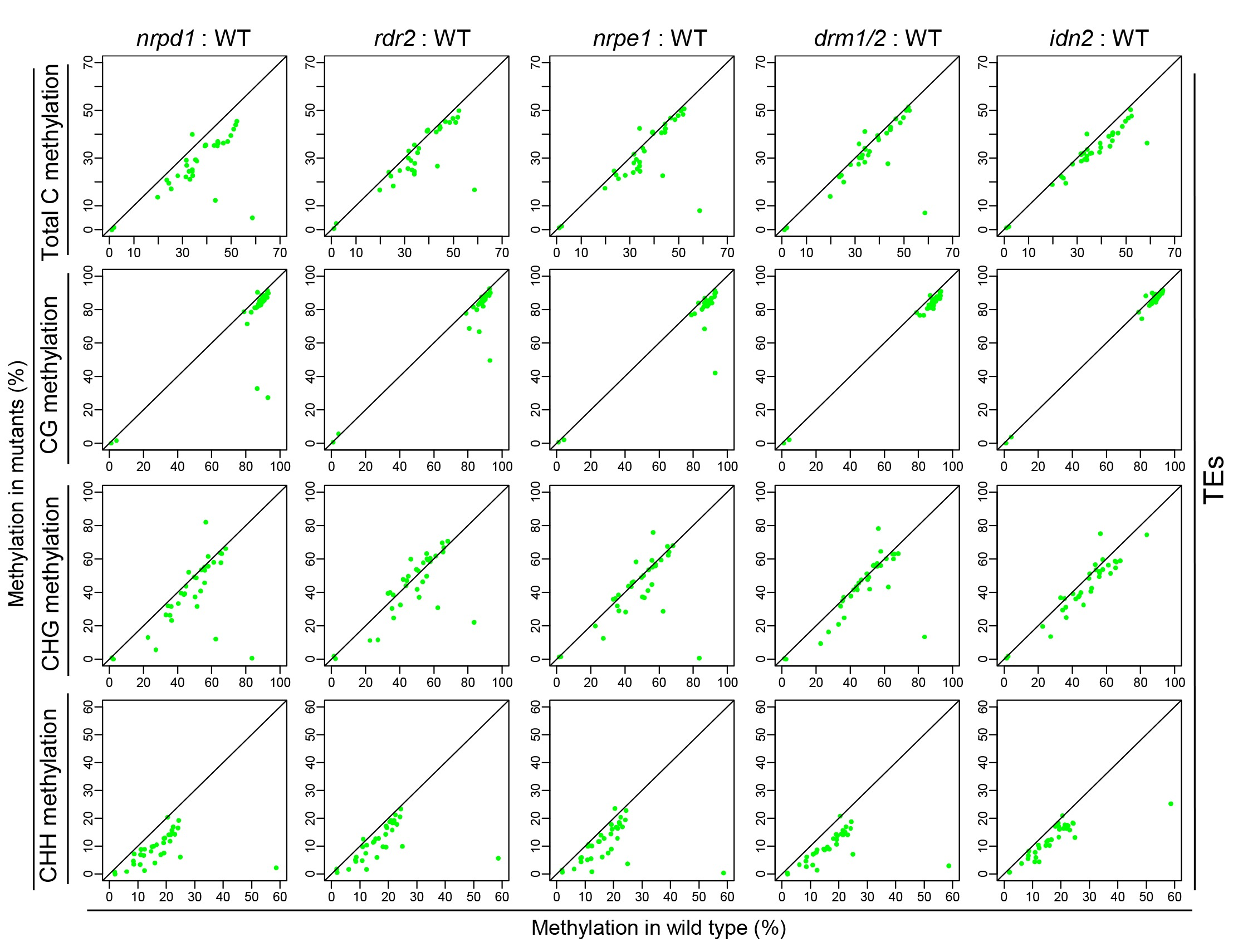

Supplement: S12 Fig — Green dots shown in the scatter plots represent total C, CG, CHG, and CHH methylation of morc6 specific up-regulated TEs in the wild type and the mutants including nrpd1, rdr2, nrpe1, drm1/2, and idn2. (TIF) [file pgen.1006026.s012.tif]

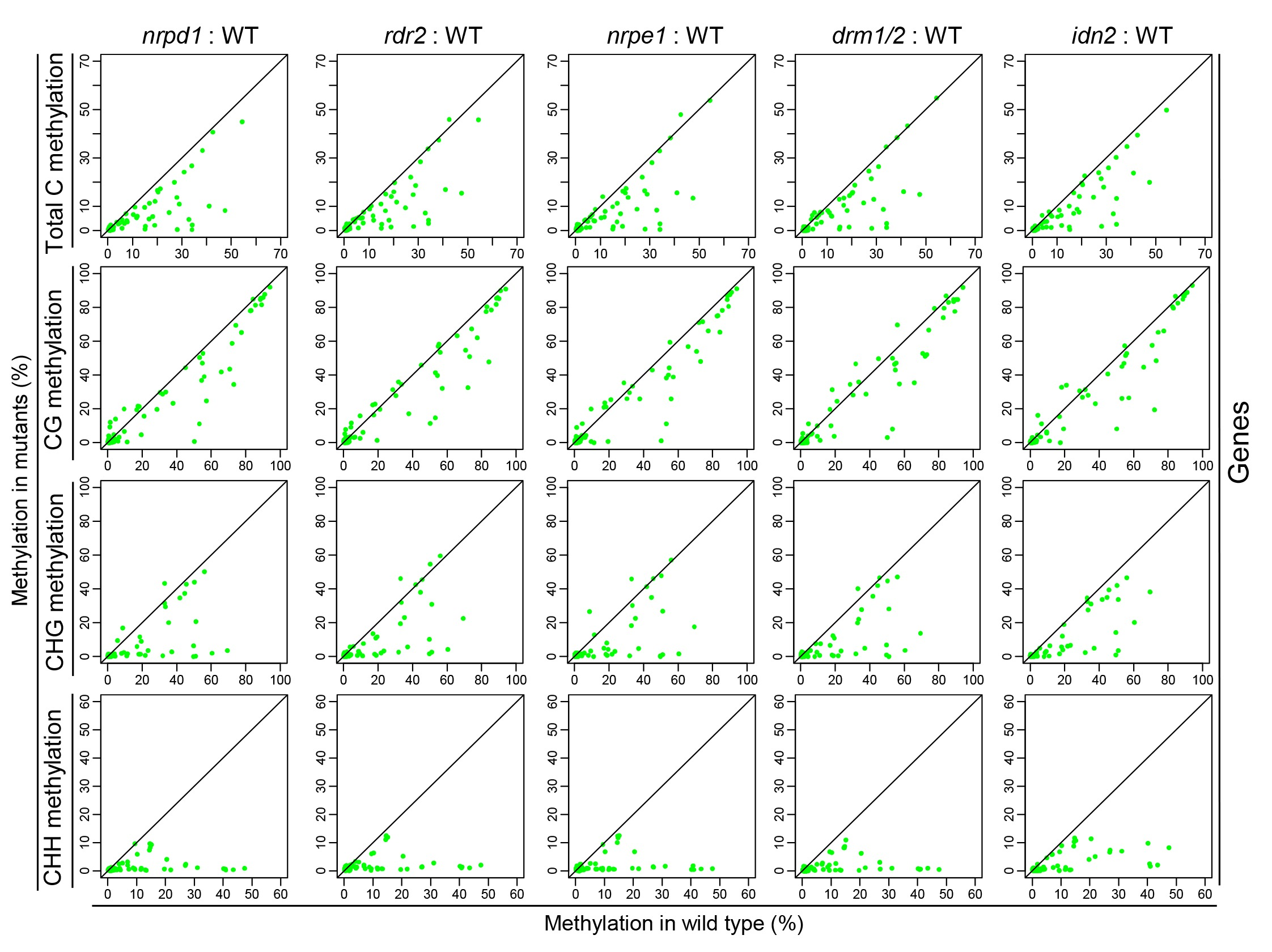

Supplement: S13 Fig — Green dots shown in the scatter plots represent total C, CG, CHG, and CHH methylation of morc6 specific up-regulated genes in the wild type and the mutants including nrpd1, rdr2, nrpe1, drm1/2, and idn2. (TIF) [file pgen.1006026.s013.tif]

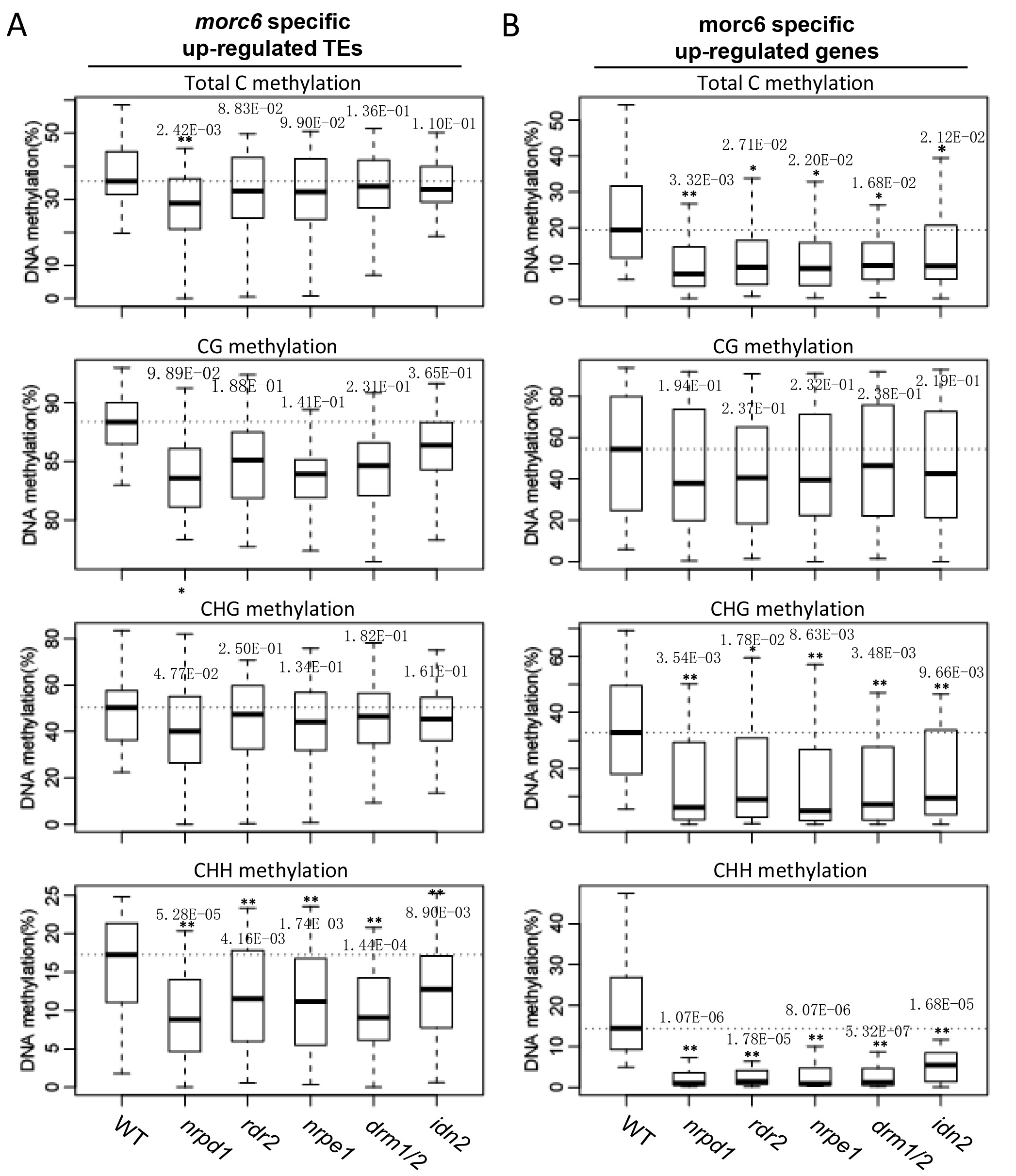

Supplement: S14 Fig — (A) DNA methylation of TEs that are specifically up-regulated in morc6. (B) DNA methylation of genes that are specifically up-regulated in morc6. Total cytosine, CG, CHG, and CHH methylation are separately indicated. Asterisks indicate statistical significance (t-test; * p<0.05, ** p<0.01). p value is shown for each sample. (TIF) [file pgen.1006026.s014.tif]

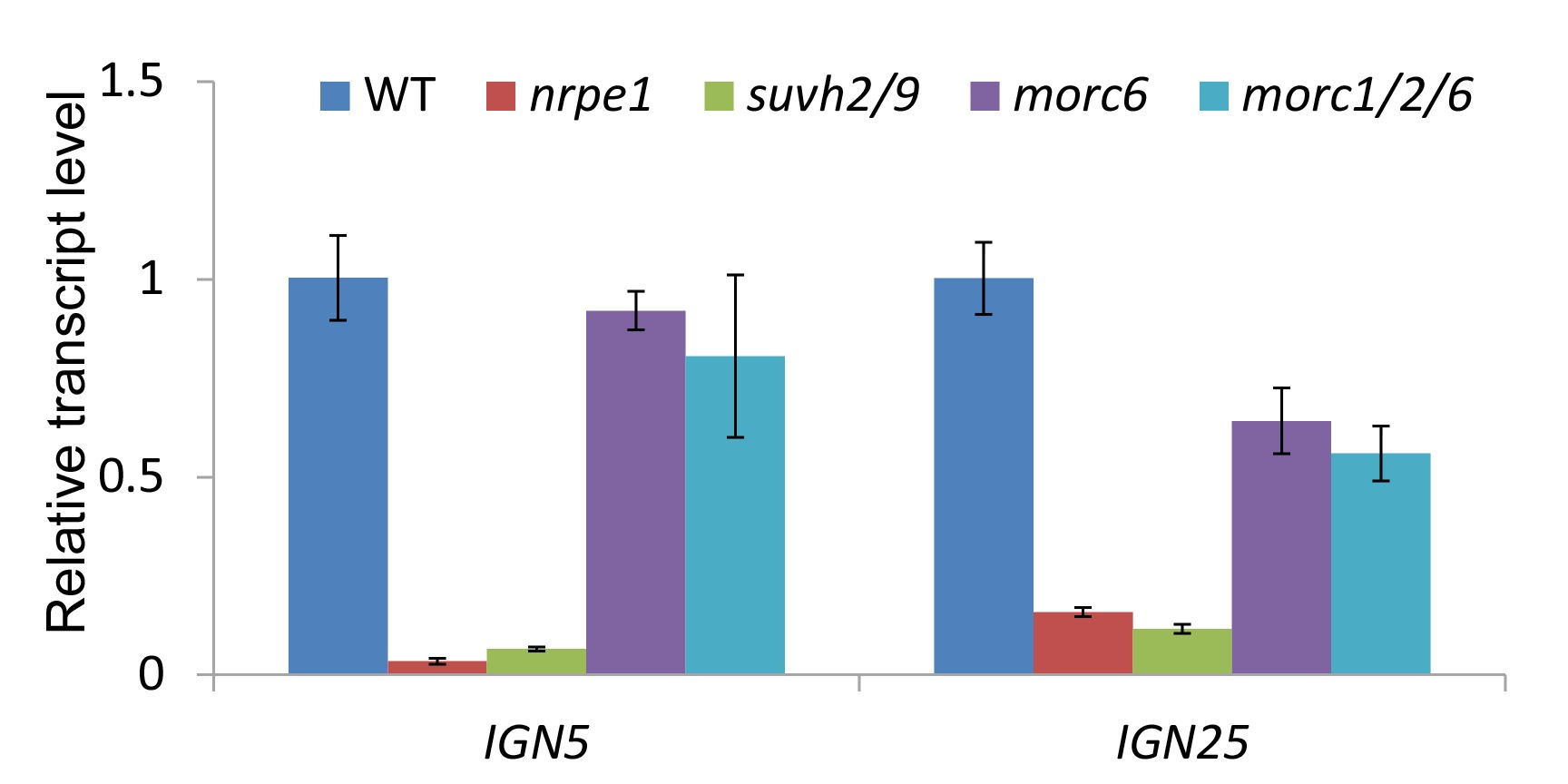

Supplement: S15 Fig — The RNA transcript levels of IGN5 and IGN25 were determined by quantitative RT-PCR in the wild type, nrpe1, suvh2/9, morc6, and morc1/2/6. The actin gene ACT2 was used as an internal control. (TIF) [file pgen.1006026.s015.tif]
